# N(alpha)-acetyltransferase 40-mediated histone acetylation plays an important role in ecdysone regulation of metamorphosis in the red flour beetle, *Tribolium castaneum*

**DOI:** 10.1038/s42003-024-06212-7

**Published:** 2024-05-03

**Authors:** Sharath Chandra Gaddelapati, Smitha George, Anilkumar Moola, Karthi Sengodan, Subba Reddy Palli

**Affiliations:** 1 Department of Entomology, College of Agriculture, Food and Environment, University of Kentucky, Lexington, KY 40546 USA; 2 Present Address: Donald Danforth Plant Science Center, St. Louis, MO 63132 USA

**Keywords:** Entomology, Animal physiology

## Abstract

Histone acetylation, a crucial epigenetic modification, is governed by histone acetyltransferases (HATs), that regulate many biological processes. Functions of HATs in insects are not well understood. We identified 27 HATs and determined their functions using RNA interference (RNAi) in the model insect, *Tribolium castaneum*. Among HATs studied, N-alpha-acetyltransferase 40 (*NAA40*) knockdown caused a severe phenotype of arrested larval development. The steroid hormone, ecdysone induced *NAA40* expression through its receptor, EcR (ecdysone receptor). Interestingly, ecdysone-induced *NAA40* regulates *EcR* expression. NAA40 acetylates histone H4 protein, associated with the promoters of ecdysone response genes: *EcR*, *E74*, *E75*, and *HR3*, and causes an increase in their expression. In the absence of ecdysone and NAA40, histone H4 methylation by arginine methyltransferase 1 (ART1) suppressed the above genes. However, elevated ecdysone levels at the end of the larval period induced *NAA40*, promoting histone H4 acetylation and increasing the expression of ecdysone response genes. NAA40 is also required for EcR, and steroid-receptor co-activator (SRC) mediated induction of *E74*, *E75*, and *HR3*. These findings highlight the key role of ecdysone-induced *NAA40*-mediated histone acetylation in the regulation of metamorphosis.

## Abstract (web summary)

The epigenetic regulator, N(alpha)-acetyltransferase 40 (NAA40), induced by ecdysone, acetylates histone H4 and modulates ecdysone action in the regulation of insect metamorphosis.

## Introduction

Epigenetic modifications regulate many fundamental biological processes by tightly regulating chromatin structure, influencing promoter access, and modulating gene expression^1,2^. Among these modifications, acetylation is one of the crucial regulatory mechanisms carried out by acetyltransferases. The diversity of acetyltransferases is remarkable, and they are categorized based on their subcellular localization as well as structural and functional similarities of their catalytic domains. Based on subcellular localization, histone acetyltransferases are divided into two classes: type A HATs located in the nucleus and type B HATs located in the cytoplasm. However, some HAT proteins function in multiple locations, making their classification challenging. To address this ambiguity, acetyltransferases are further categorized into different families based on structural and functional similarities of their catalytic domains. For instance, lysine acetyltransferases (KATs) are known for adding an acetyl moiety to the epsilon-amino group of lysine residues in histones and other proteins^3,4^. Within KATs families, GCN5-related N-acetyltransferases (GNAT), MYST and p300/CBP families are extensively studied in mammals^5,6^. GNAT catalyze the transfer of an acetyl group from acetyl-coenzyme A (Ac-CoA) to various primary amine substrates, including histones and these are the first identified KATs^7^. The MYST family KATs acetylates both histones and non-histone proteins, regulating diverse functions including gene regulation, DNA repair, cell cycle, stem cell homeostasis and development^8^. The another KAT family member - CREB-binding protein (CBP) emerged as a key player in juvenile hormone (JH) signaling, specifically by inducing the JH primary response gene, Krüppel homolog 1 (*Kr-h1*) in both Red flour beetle, *Tribolium castaneum* and Yellow fever mosquito, *Aedes aegypti* in our previous studies^9–12^. Conversely, histone deacetylases (HDACs), especially HDAC1, HDAC3, and HDAC11, exhibited contrasting effects by removing acetyl groups from lysine amino acids in histones and repressing JH signaling by suppressing the *Kr-h1* gene^13–15^. These studies highlight the critical roles of histone acetylation and deacetylation in insect hormone actions that regulate insect development. In the model insect, *Drosophila melanogaster*, several KATs have been identified and characterized, however, their functional characterization in other insect species is lacking.

Another class of acetyltransferases, N-terminal acetyltransferases (NATs) mediate widespread protein modification that is conserved from yeast to humans. NATs transfer the acetyl group from Ac-CoA to the α-amino group in the N-terminal amino acid residue of histone proteins or peptides. NATs play crucial roles in multiple biological processes, including protecting proteins from degradation, proteasome localization, cell survival, hormonal regulation, apoptosis and maintenance of organelle structure, and function^2,16–21^. In humans, six NATs (NatA-NatF) have been identified, each with unique subunit composition, substrate preferences, and induced phenotypes^2,22^. However, information on NATs functions in insects, except in *Drosophila*, is limited. This knowledge gap presents an opportunity to investigate the roles of acetyltransferases in other insects beyond *Drosophila*.

In this study, we identified various acetyltransferases potentially involved in the growth, development, and metamorphosis of the model insect, *T. castaneum*. To determine the functions of these acetyltransferases, we performed RNA interference (RNAi) experiments to knockdown genes coding for KATs and NATs in *T. castaneum*. Among the acetyltransferases studied, the knockdown of *NAA40* (TC015921), a member of the NAT family showed severe phenotypes, including larval development arrest and mortality during metamorphosis. This observation led us to prioritize NAA40 for further in-depth investigations to comprehend its role in regulating insect hormone action, development, and metamorphosis.

## Results

### Identification and determination of functions of acetyltransferases in the red flour beetle, *T. castaneum*

We identified 27 genes coding for acetyltransferases in *T. castaneum* genome using *D. melanogaster* HAT protein sequences. These HATs were classified into Lysine acetyltransferases (KATs) and N-terminal acetyltransferases (NATs) based on the structural and functional similarities of their catalytic domains. Among the 27 HATs identified, 12 belong to the KATs category (Supplementary Table 1). Within in the KATs category, KAT5, KAT6A, KAT7 and KAT8 were identified to contain the MYST domain, known for its involvement in the acetylation of many nuclear proteins that regulate many biological functions, including gene regulation, DNA repair, cell-cycle regulation, apoptosis, and development^8^. Previous studies have already identified two multifunctional KATs, CREB binding protein (CBP), and steroid receptor co-activator (SRC), as important players in the hormonal regulation of development and metamorphosis in *T. castaneum*^9–11,23^.

**Fig. 1:**
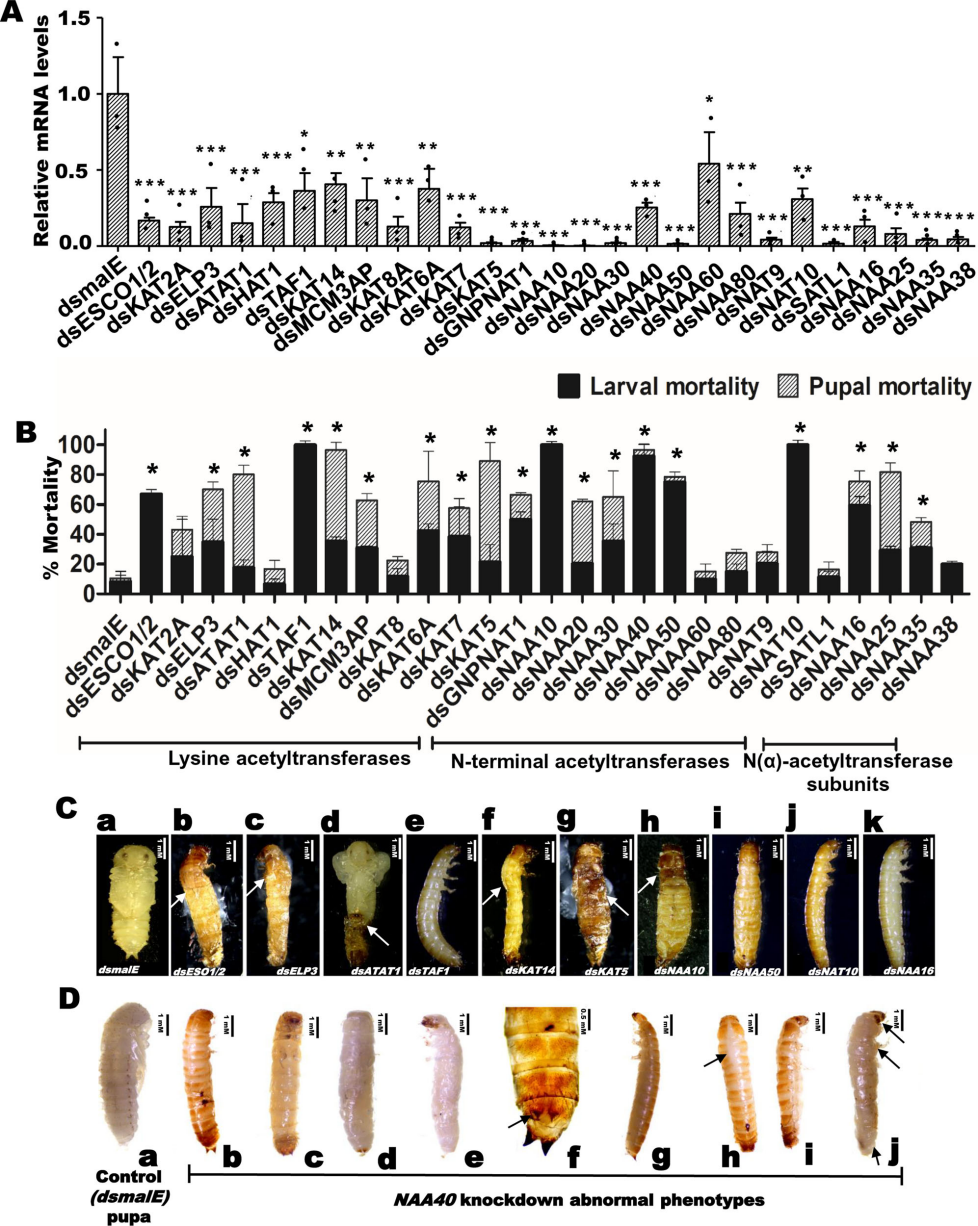
Knockdown of acetyltransferases caused mortality and phenotype changes in *T. castaneum.* **A** Knockdown levels of various acetyltransferases in larvae injected with cognate dsRNAs. Twelve lysine acetyltransferases (KATs), eleven N-terminal acetyltransferases (NATs) and four N-α-acetyltransferases (NAAs) subunits were selected for preliminary screening in this study. Newly molted last instar larvae were injected with cognate dsRNAs targeting the above acetyltransferases. Control insects were injected with dsRNA targeting gene encoding for maltose-binding protein from *Escherichia coli* (malE). Knockdown levels of target genes at 72 h post-injection of cognate dsRNA were determined by RT-qPCR and normalized with the Ribosomal Protein 49 (*RP49*) mRNA levels. The results presented as Mean ± SE (*n* = 4), reveal significant differences (***P < 0.001; **P < 0.01; *P < 0.05) in the target gene expression levels in treatments compared to the control treated with *dsmalE*, analyzed using the One-way ANOVA. **B** Knockdown of acetyltransferases caused larval and pupal mortality in *T. castaneum*. Mortality was recorded every day until death or adult eclosion. Mean + SE (*N* = 90) from three independent experiments showed. ‘*’ denotes significant differences in the mortality of larvae and pupae in treatments compared to that in the control analyzed using the One-way ANOVA at *P* < 0.005. Information about each gene is provided in Supplementary Tables 1−3. **C** Larval or pupal phenotypes after dsRNA injection: (**a**) control larva injected with *dsmalE* pupated with properly folded wings and later emerged as a normal adult. **b** *dsESCO1/2* injected larvae showed split in the dorsal thoracic region (dorsal split-white arrow) and sclerotized head and they were not able to complete molting. **c** *dsELP3* injected larvae showed dorsal split (white arrow) and sclerotized head though they did not molt into the next stage. **d** *dsATAT1* injected larvae developed into defective winged pupae (exuviae remain attached to the body – indicated with white arrow). **e** *dsTAF1* injected larvae died during the quiescent stage. **f** *dsKAT14* injected larvae showed dorsal split (white arrow), sclerotized head and legs. **g** *dsKAT5* injected larvae developed into larval-pupal intermediaries and died during the pupal stage. **h** *dsNAA10* injected larvae developed into larvae-pupal intermediaries with sclerotized head and prothoracic region (white arrow). **i** *dsNAA50* injected larvae died during the quiescent stage. **j** *dsNAT10* injected larvae died during the quiescent stage. **k** *dsNAA16* injected larvae died before the quiescent stage. **D** *dsNAA40* injected larvae stayed in larval stage for longer period. **a** Control pupa injected with *dsmalE*. **b**, **c** Larval phenotype without the dorsal split. **d**, **e** After the removal of the cuticle, the white-colored larval phenotype was observed inside. **f** Additional larval urogomphi (black arrow) developed in the posterior region of larva. **g** Wild-type last instar larva. **h**, **i** The larval-pupal intermediate phenotype of *dsNAA40* injected larvae with dorsal split (black arrow). After nine days of treatment, insects developed a dorsal split. **j** After removal of the cuticle, the white-colored larval-pupal-adult intermediate phenotype was found with compound eyes, pupal urogomphi and gin-traps. Scale bar: 1 mM.

To characterize the function of identified acetyltransferases, we knockdown all 27 acetyltransferases and evaluated knockdown efficiency in larvae injected with their respective cognate dsRNA. The mRNA levels of most of the target genes exhibited a reduction of over 70% in respective dsRNA treatments when compared to their mRNA levels in control larvae injected with *dsmalE* (Fig. 1A and Supplementary Data 2). Then we recorded the phenotypic changes resulting from the knockdown of each acetyltransferase. Among the KATs studied, knockdown of Transcription initiation factor IID (TFIID) subunit 1 (*TAF1*) caused 100% larval mortality by 10 days after dsRNA injection (Fig. 1B). Developmental growth defects and mortality were observed in larvae and pupae developed from larvae injected with *dsESCO1*/*2*, *dsELP3* (Elongator complex protein 3), *dsATAT1* (Alpha-tubulin N-acetyltransferase 1), *dsKAT14* (Atac2, Cysteine-rich protein 2-binding protein) and *dsKAT5* (Tip60) (Fig. 1C). Moreover, moderate to higher mortality was observed in larvae injected with *dsKAT2A*, *dsMCM3AP*, *dsKAT6A*, and *dsKAT7* compared to the control (Fig. 1B). Eleven genes coding for N-terminal acetyltransferases (NAT) and four genes coding for N (alpha)-acetyltransferase (NAA) subunits were identified in *T. castaneum*, respectively (Supplementary Tables 2 and 3). Among the NAA subunit coding genes, knockdown of *NAA16* arrested larval growth and caused 60% larval mortality, while the knockdown of *NAA25* and *NAA35* also caused developmental defects and mortality (Fig. 1B, C). Among the NAT group, the knockdown of *NAA10* (N-alpha-acetyltransferase 10), *NAA40* (N-alpha-acetyltransferase 40), *NAA50* (N-alpha-acetyltransferase 50), and *NAT10* (RNA cytidine acetyltransferase) caused severe developmental defects and higher larval mortality (Fig. 1B, C). Similarly, the knockdown of *GNPNAT1* (Glucosamine 6-phosphate N-acetyltransferase), *NAA20* (N-alpha-acetyltransferase 20), *NAA30* (N-alpha-acetyltransferase 30), and *SATL1* (Diamine acetyltransferase 2) caused developmental defects, as well as larval and pupal mortality. Notably, the most severe phenotypes such as larval growth arrest at the last instar larval stage, dorsal split failure, inability to pupate, and high larval mortality, were observed in the *NAA40* knockdown larvae. Given these compelling observations, our further studies were focused on understanding the precise role of NAA40 in regulating larval and pupal growth, development, and metamorphosis in *T. castaneum*.

### NAA40 is essential for larval growth, development, and metamorphosis

To investigate the role of NAA40 (NatD) in the development and metamorphosis of *T. castaneum*, we injected 1 µg of *dsNAA40* or *dsmalE* (negative control) into newly molted last instar larvae and recorded phenotype changes. The results revealed that *NAA40* knockdown larvae remained struck in larval stage and failed to form complete pupae even after nine days, whereas the control larvae injected with *dsmalE* pupated within seven days. Notably, larvae did not develop a dorsal split, but a white larval cuticle was detected upon removing the old cuticle on the 9th day after *dsNAA40* injection (Fig. 1D: b−e). Then these larvae transformed into larval-pupal intermediates, displaying characteristics such as dorsal splitting and the presence of pupal and adult structures (Fig. 1D: h, i). We further verified the phenotypes resulting from *NAA40* knockdown by injecting the last instar larvae with various concentrations: 50, 100, 250, 500 ng and 1 µg of *dsNAA40*. Notably, the higher concentrations (500 ng and 1 µg) of *dsNAA40* led to higher knockdown of target gene, increased mortality, and severe developmental defects, including formation of larval-pupal intermediates with compound eyes. Lower concentrations (50, 100 and 250 ng) of *dsNAA40* injection resulted in moderate phenotypes compared to the 1 µg *dsNAA40* treatment (Supplementary Fig. 1). To understand the effects of *NAA40* knockdown at different instars, we injected 1 µg of *dsNAA40* into penultimate larvae. These larvae showed dorsal split after six days (Fig. 1D: h, i), and some larvae molted into the last instar stage, then larval growth arrest occurred at 13 days after dsRNA injection. Also, pupal development was arrested when *dsNAA40* was injected into the newly formed pupae. Contrastingly, the control group consisting of newly molted last instar larvae, penultimate larvae, or pupae injected with *dsmalE* displayed normal growth and development, they pupated and later emerged as normal adults (Fig. 1C_a, D_a).

### Ecdysone induces *NAA40* expression

**Fig. 2:**
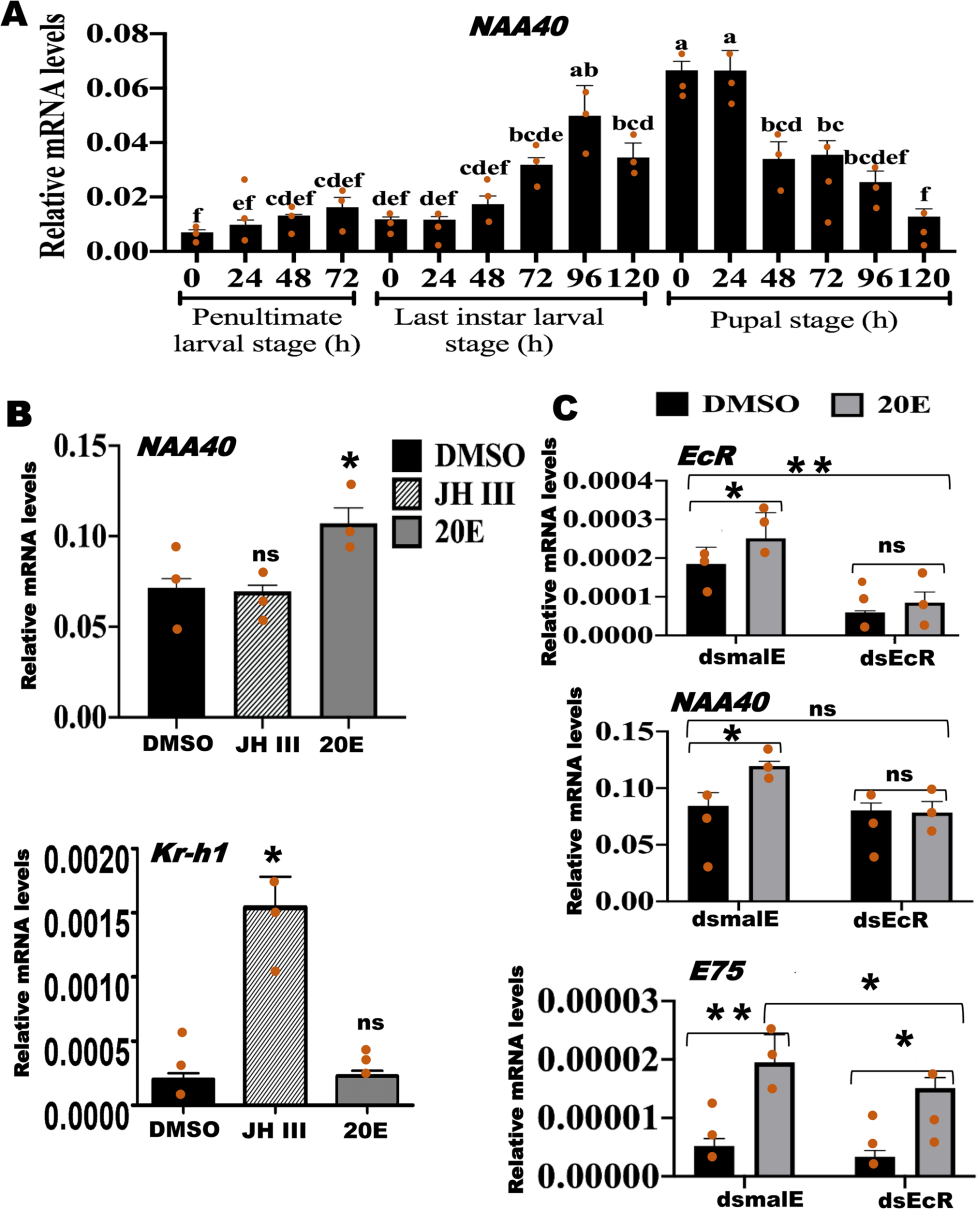
*NAA40* is induced by ecdysone through its receptor, EcR. **A** Developmental expression profiles of *NAA40* in *T. castaneum* determined by RT-qPCR and normalized with the Ribosomal Protein 49 (*RP49*) mRNA levels. Samples were collected at 24 h intervals during the penultimate, last instar larvae, and pupal stages. For each time, two larvae/pupae were used for one replication. The mean ± SE of three replications is shown. Bars with the same letters are not significantly different from each other at *P* < 0.05. **B** Ecdysone inudces *NAA40* expression in *T. castaneum* cell line - TcA. While the *Kr-h1*, a primary JH response gene, was employed as a positive control to assess the activity of juvenile hormone III (JH III). The *NAA40* and *Kr-h1* mRNA levels were determined using the total RNA isolated from TcA cells exposed to 10 μM of 20-hydroxyecdysone (20E) or JH III or DMSO. **C** EcR is required for ecdysone induction of *NAA40*. TcA cells were exposed to *dsEcR* or *dsmalE*. At 72 h post-dsRNA treatment, the cells were treated with DMSO or ecdysone for 6 h. Total RNA isolated from cells was used to quantify *EcR*, *NAA40*, and *E75* mRNA levels. The data shows mean ± SE of three replicates. Asterisks ‘*or **’on bars indicates significant differences in the target gene expression levels in treatments compared to the control treated with *dsmalE* at *P* < 0.05 and *P* < 0.01, respectively analyzed using the One-way ANOVA with Post hoc Tukey HSD test. Here, ns, not significant.

To understand the role of NAA40, we determined the expression profile of *NAA40* during the larval and pupal developmental stages of *T. castaneum*. The results revealed that *NAA40* mRNA levels gradually increased from the penultimate larval stage, reaching maximum at 24 h after pupal ecdysis. Then they decreased and reached their lowest levels by the end of the pupal stage (Fig. 2A and Supplementary Data 2). The expression pattern of *NAA40* exhibited a similar trend to the ecdysone titer levels during the penultimate and last instar larval and pupal stages of *T. castaneum*^24^. Intrigued by this similar pattern, we investigated whether *NAA40* is induced by ecdysone. In this regard, we exposed TcA cells (developed from *T. castaneum*) to 10 µM of 20-hydroxyecdysone (20E-most active form of ecdysone, hereafter referred to as ecdysone) for 6 h and determined the *NAA40* expression. The results showed that *NAA40* mRNA levels increased in ecdysone-treated cells compared to control cells exposed to Dimethyl sulfoxide (DMSO) (Fig. 2B). However, exposure to juvenile hormone III (JH III) did not induce the expression of the *NAA40* gene. While, the JH primary response gene, *Kr-h1* used as a positive control was induced by JH III in these cells, demonstrating the activity of JH III used in these experiments (Fig. 2B). Moreover, ecdysone typically induces its target genes through its receptor known as ecdysone receptor (EcR)^25^. To determine whether EcR is required for the ecdysone induction of *NAA40*, we treated TcA cells with *dsEcR* or *dsmalE* for 72 h before exposure to ecdysone or DMSO. As expected, *NAA40* mRNA levels increased in TcA cells treated with *dsmalE* and exposed to ecdysone but not in *EcR* knockdown cells exposed to ecdysone (Fig. 2C). To confirm the effect of ecdysone on these cells, we examined the expression of *E75*, an ecdysone-induced transcription factor, serving as a positive control, which was induced by ecdysone (Fig. 2C).

### Knockdown of *NAA40* decreases the expression of ecdysone-response genes

**Fig. 3:**
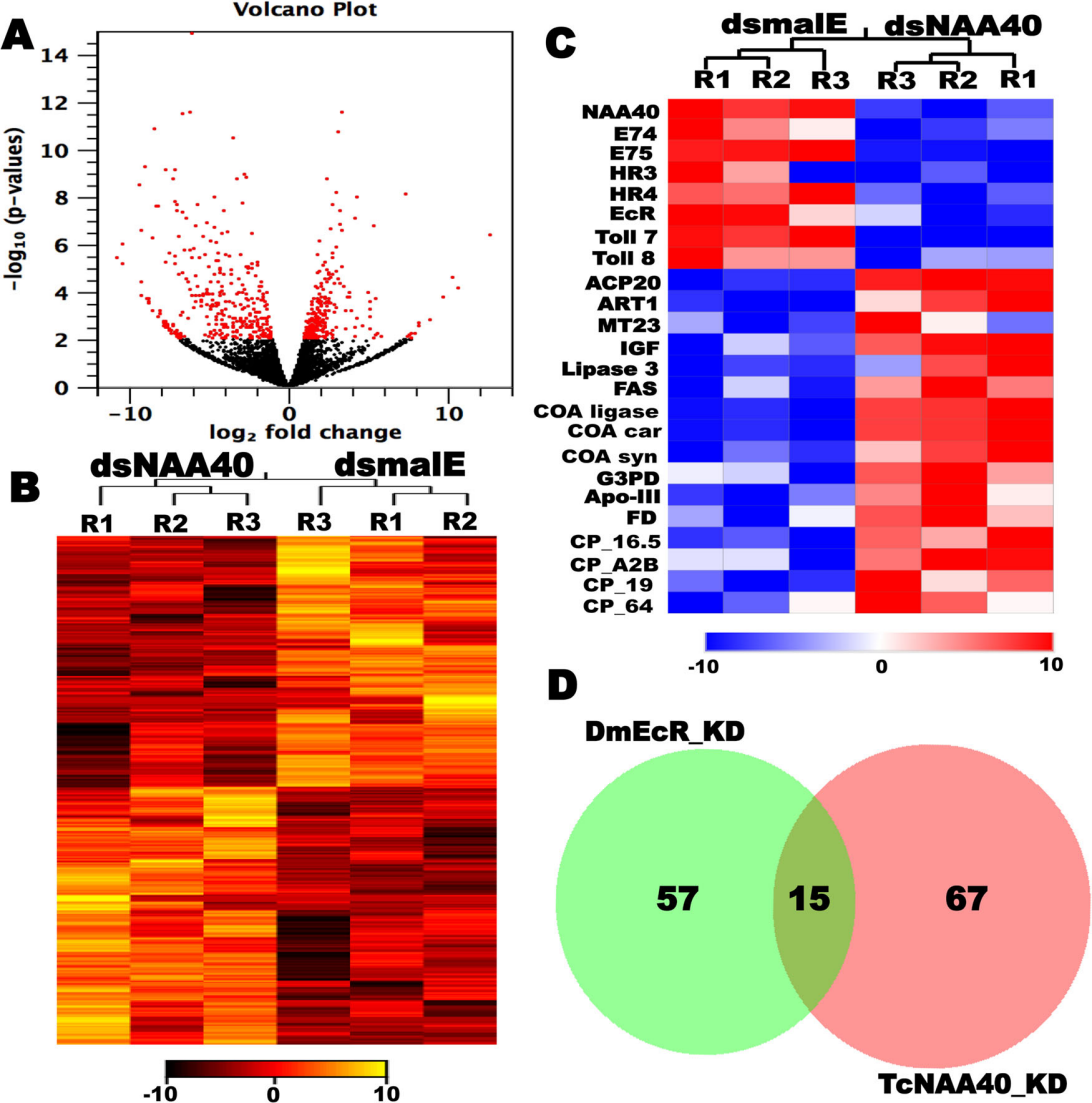
*NAA40* knockdown affects ecdysone response genes expression. **A** Volcano plot represents expression data after EDGE analysis. In the plot, log2 fold change between the *dsmalE* and *dsNAA40* treated insects are plotted against −log_10_ *P* values. The red dots indicate the number of significantly up- and down-regulated genes identified after implying false discovery rate (FDR) corrected *P* < 0.05 and ±2-fold change criteria. **B** The heatmap shows TMM normalized, log_2_ transformed expression values of differentially expressed 731 genes (FDR corrected *p* ≤ 0.05 and ≥2-fold) in the *NAA40* knockdown larvae. **C** The heatmap shows the differential expression of some key genes affected by the *NAA40* knockdown. The heatmap represents the normalized and log_10_ transformed values of key downregulated and upregulated genes expression levels in *NAA40* knockdown larvae compared to control larvae treated with *dsmalE*. Here, NAA40: N-alpha-acetyltransferase 40, E74: Ecdysone-induced protein 74EF, E75: Ecdysone-inducible protein E75, HR3: Hormone receptor 3, HR4: Hormone receptor 4, EcR: Ecdysone receptor, Toll7: Toll-7-like protein, Toll8: Toll-like receptor 8, ACP20: Adult-specific cuticular protein 20, ART1: Arginine N-methyltransferase 1, MT23: Methyltransferase-like protein 23, IGF: Insulin-like growth factor, FAS: Fatty acid synthase, COA ligase: Fatty-acid-CoA ligase, CoA Car: Acetyl-CoA carboxylase, CoA Syn: Acetyl-CoA synthase, G3PD: Glyceraldehyde 3-phosphate dehydrogenase, Apo-III: Apolipophorin-III, FD: Farnesol dehydrogenase, CP_16.5: Cuticle Protein 16.5, CP_A2B: Cuticle Protein A2B, CP_19: Cuticle Protein 19, CP_64: Cuticle Protein 64. **D** Knockdown of *TcNAA40* or *DmEcR* affects some common gene ontologies. The GO terms of *NAA40* knockdown RNA-seq data compared with the GO terms of the *EcR* knockdown data from *Drosophila*. A list of common gene ontologies affected by both *TcNAA40* and *DmEcR* knockdown is provided in Supplementary Table 4.

Next, to investigate the role of ecdysone-induced *NAA40* in the modulation of ecdysone-response genes, we knocked down *NAA40* in *T. castaneum* larvae by injecting *dsNAA40* or *dsmalE*. RNA isolated from treated larvae at 72 h post-injection was used to prepare RNA-sequencing libraries and which were then sequenced. After quality control, the mapping of sequencing reads indicated that 90% of the reads were mapped to the *T. castaneum* reference genome. Subsequent differential expression analysis of RNA-seq data identified 731 differentially expressed genes (DEGs) between *dsNAA40* and *dsmalE* treated larvae, with a cutoff value ≥ 2-fold difference in the expression and false discovery rate (FDR) corrected *P* ≤ 0.05 (Supplementary Data 1). Among these DEGs, 55% (401) genes were upregulated, and 45% (330) genes were downregulated in *NAA40* knockdown larvae (Fig. 3A, B and Supplementary Data 1). Web-based gene enrichment (WEGO) analysis of differentially expressed genes showed significant alterations in GO terms related to circadian rhythm, anatomical structure development, response to external or endogenous stimuli, signal transducers, and ecdysone response genes (Supplementary Fig. 2). Notably, genes involved in lipid biosynthesis, larval cuticle development and methylation related methyltransferases, such as arginine N-methyltransferase 1 (ART1) and methyltransferase-like protein 23 (MTP23), were upregulated in the *NAA40* knockdown samples (Fig. 3C). Interestingly, the expression levels of many ecdysone response genes, including *E74*, *E75* and *HR3*, were decreased in the *NAA40* knockdown samples indicating that NAA40 is required for ecdysone signaling. To determine if NAA40 exerts its influence on ecdysteroid biosynthesis, we assessed the expression levels of Shadow and Phantom genes involved in ecdysteroids biosynthesis in *NAA40* knockdown larvae. Intriguingly, the expression levels of both *Shadow* and *Phantom* genes did not change in the *NAA40* knockdown last instar larvae compared to their expression levels in control larvae injected with *dsmalE* (Supplementary Fig. 3). This result indicates that NAA40 exerts its influence on ecdysone signaling rather than influencing ecdysteroid biosynthesis. Ecdysone regulates the transcription of ecdysone response genes through ecdysone receptor (EcR) complex. Therefore, to elucidate whether NAA40 is involved in ecdysone signaling, we compared *NAA40* knockdown RNA-seq data with ecdysone receptor (*EcR*) knockdown data from *Drosophila*^25^. Interestingly, knockdown of *TcNAA40* or *DmEcR* affected some common gene ontologies, such as cuticle development, lipid transport, gluconeogenesis, neuropeptide signaling, and steroid hormone mediated signaling, etc. (Fig. 3D and Supplementary Table 4). Therefore, these results indicate a potential connection between NAA40 and ecdysone signaling.

### NAA40 acetylates histone H4

**Fig. 4:**
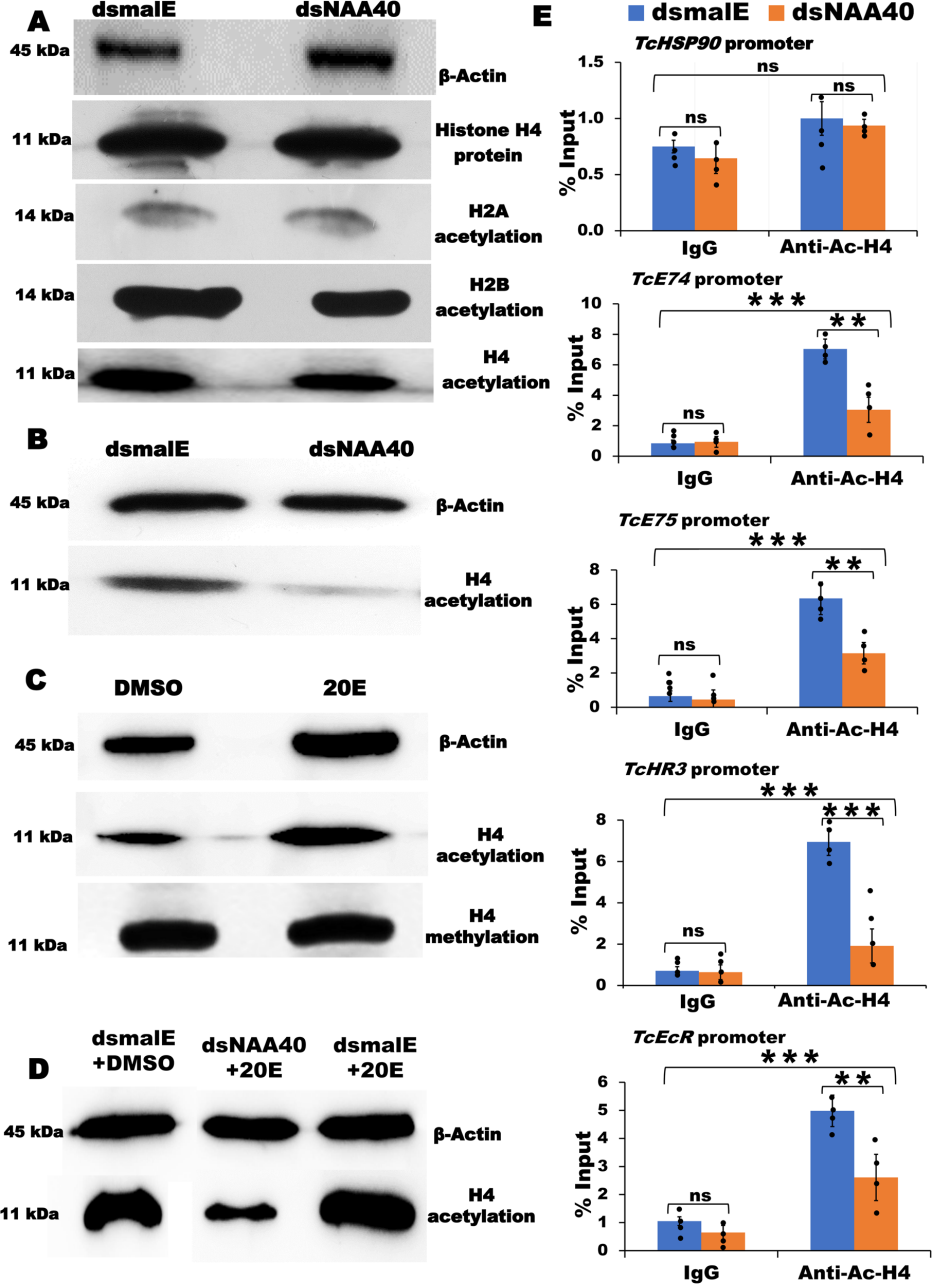
Ecdysone-induced *NAA40* acetylates histone H4 associated with *E74*, *E75* and *HR3* promoters. **A** Knockdown of *NAA40* decreased the acetylation levels of histone H4 but had no effect on the histone H4 protein levels and acetylation levels of histone H2A and H2B in TcA cells. **B** Histone H4 acetylation levels reduced after *NAA40* knockdown in *T. castaneum* larvae detected using the Ac-Histone H4 antibody. **C** Ecdysone effect on the histone H4 acetylation and methylation. TcA cells were treated with ecdysone and assessed histone H4 acetylation (using the H4-specific Ac-Histone H4 antibody) and methylation (using the Anti-Histone H4 asymmetric di-methyl Arg3 antibody). **D** *NAA40* knockdown prevented ecdysone-mediated induction of histone H4 acetylation levels in TcA cells. Target protein acetylation/methylation levels among treatments and control were normalized using the loading control protein, β-Actin. **E** Chromatin immunoprecipitation (ChIP) assay showed that *NAA40* knockdown significantly reduces the enrichment levels of ecdysone receptor (*EcR*), *E74, E75* and *HR3* promoters in TcA cells. *HSP90* promoter enrichment levels were used as a control. The rabbit IgG antibody immunoprecipitated chromatin served as a negative control. Each immunoprecipitated sample normalized with its input material and promoter enrichments were represented as percent input. Data are shown mean ± SE of four replicates. Asterisks ‘** or ***’ on bars indicates significant differences in the target gene expression levels between treatments compared to the control treated with *dsmalE* at *P* < 0.01 and *P* < 0.005, respectively analyzed using ANOVA with Post hoc Tukey’s honestly significant difference (HSD) test. Here, ns, not significant.

NAA40, an N-terminal acetyltransferase acetylates histone H2A/H2B and H4 to activate target genes^26,27^. To identify which histone proteins are acetylated by NAA40, we knocked down *NAA40* in TcA cells for 72 h. The nuclear proteins isolated from these cells were used to perform western blots with antibodies specific to individual acetylated histones. Surprisingly, the histone H2A and H2B acetylation levels did not show significant changes in the *NAA40* knockdown cells. However, a significant decrease was observed in the acetylation levels of histone H4 in TcA cells after *NAA40* knockdown (Fig. 4A and Supplementary Figs. 5, 11). This decrease was detected using the histone H4 specific Ac-Histone H4 antibody, which detects acetylation of Ser1, Lys5, Lys8, and Lys12 residues in the histone H4 tail. To further validate these findings in vivo, we reconfirmed the reduction in histone H4 acetylation levels in *T. castaneum* larvae after *NAA40* knockdown (Fig. 4B). These results together demonstrate that NAA40 acetylates histone H4. Further, to identify which residue of histone H4 is acetylated by NAA40, we performed western blotting using the histone H4-residue specific antibodies that detect acetylation of Lys5 or Lys8 or Lys12 in the histone H4 tail. Results revealed that the acetylation levels of Lys5, Lys8, and Lys12 residues of histone H4 were not significantly changed in the *NAA40* knockdown cells compared to their levels in the control cells treated with *dsmalE* (Supplementary Fig. 4 and Supplementary Data 2). No antibodies are available commercially to detect the histone H4 serine 1 acetylation levels. Previous literature demonstrated that NAA40 acetylates the first serine residue of histone H4 tail^27,28^. Therefore, based on these studies we presume that NAA40 may acetylate the first serine (Ser1) residue in the N-terminal region of histone H4 to alter target gene expression. However, further studies are needed to confirm the specificity of NAA40 in acetylating serine 1 residue of histone H4.

To understand the influence of ecdysone on histone H4 acetylation levels, TcA cells were exposed to ecdysone for 24 h, then nuclear proteins were extracted for assessing histone H4 acetylation levels by western blotting. Results revealed that the histone H4 acetylation levels increased in cells exposed to ecdysone compared to cells exposed to DMSO (Fig. 4C). To further delve into the role of NAA40 in ecdysone-mediated histone H4 acetylation, we knockdown *NAA40* in TcA cells and exposed them to ecdysone. Notably, the ecdysone induced histone H4 acetylation levels in TCA cells treated with *dsmalE* and exposed to 20-hydroxyecdysone (20E), compared to cells treated with *dsNAA40* and exposed to 20E (Fig. 4D). This result indicates that ecdysone mediates the induction of histone H4 acetylation levels through *NAA40*.

To investigate the potential effect of decreased histone H4 acetylation levels in *NAA40* knockdown cells on the accessibility of target gene promoters (identified based on RNA-seq data), we performed the Chromatin Immunoprecipitation (ChIP) assay. Chromatin was extracted from *NAA40* knockdown TcA cells and immunoprecipitated using the Ac-Histone H4 specific antibody. Using DNA recovered from immunoprecipitation, we analyzed the enrichment levels of target ecdysone response gene promoters, as well as the *HSP90* (housekeeping gene – control) promoter in *NAA40* knockdown cells. Knockdown of *NAA40* resulted in reduced enrichment levels of *E74*, *E75* and *HR3* gene promoters, compared with the corresponding promoter enrichment levels in the control cells treated with *dsmalE* (Fig. 4E). We did not observe any significant enrichment of the *HSP90* promoter in the *NAA40* knockdown cells. These results demonstrate that NAA40 acetylates histone H4 which may be associated with the promoters of *E74*, *E75* and *HR3* genes.

**Fig. 5:**
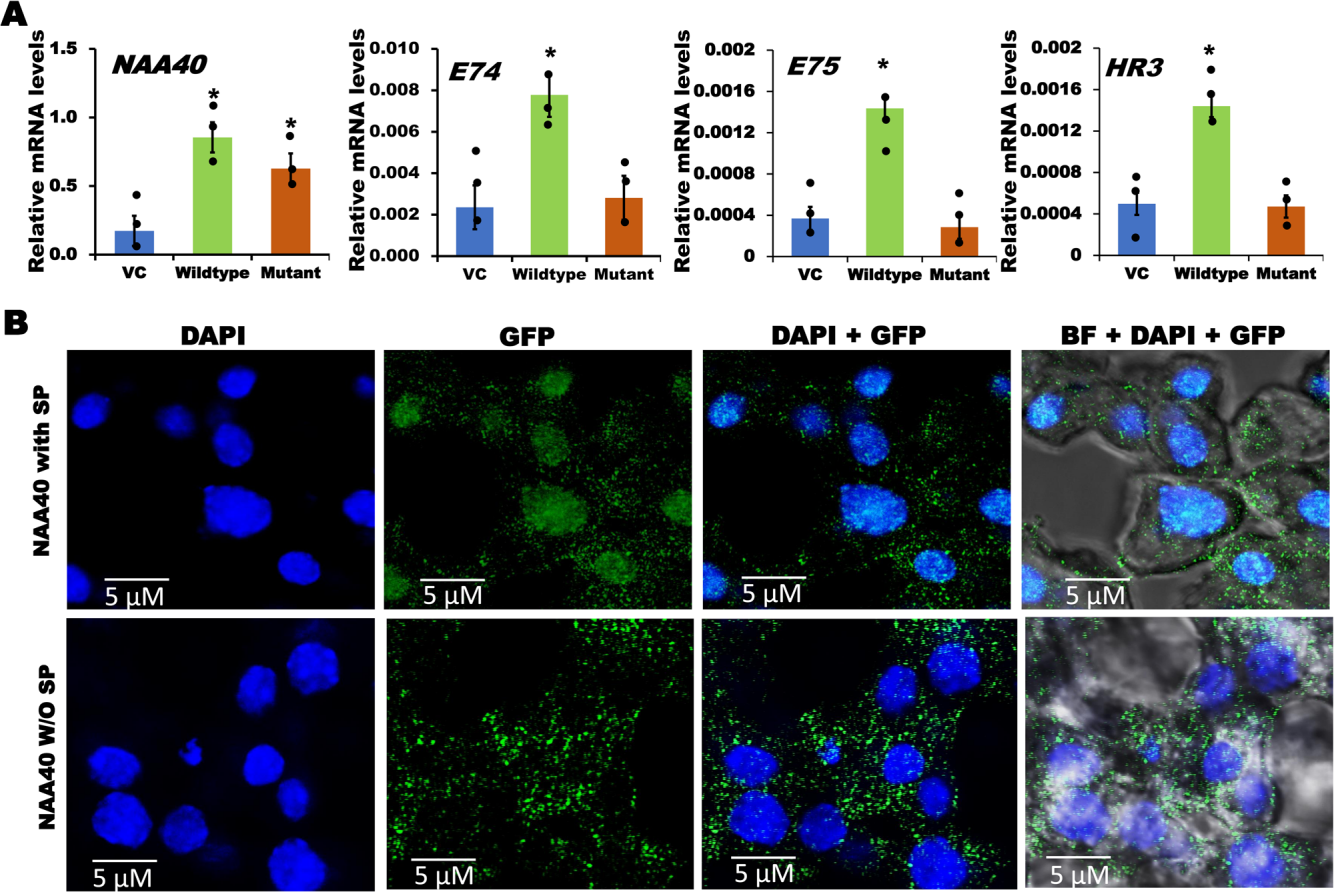
Wildtype NAA40 localizes into nucleus and may induce the expression of ecdysone response genes. **A** Over-expression of *NAA40* wild-type and mutant (lacking the acetyl-CoA binding motif) constructs differentially regulate the expression of *E74*, *E75* and *HR3* genes. The data shows mean ± SE (*n* = 3). Asterisk ‘*’ on bars indicates significant differences in the target gene expression levels between treatments and control transfected with empty vector (VC) at *P* < 0.05 analyzed using the One-way ANOVA. **B** Exogenously expressed *NAA40* localized in the nucleus to alter the histone H4 acetylation. TCA cells were transfected with wildtype *NAA40-EGFP* and N-terminal signal peptide lacking *NAA40-EGFP* fusion constructs. At 72 h post transfection, cells were mounted using the DAPI nuclear staining and imaged under Confocal microscope at 63X magnification using blue and GFP filters. The images display DAPI alone, GFP alone, both merged, as well as merged with bright field (BF). Here: NAA40 with SP: Signal peptide containing wildtype *NAA40*-EGFP fusion construct, NAA40 W/O SP: N-terminal signal peptide lacking *NAA40-EGFP* fusion construct. Scale bar 5 μm for all images.

NAA40, an N-terminal acetyltransferase, contains a conserved acetyl-CoA binding motif responsible for transferring the acetyl group from the substrate, Acetyl-CoA, to histones. To determine whether acetyltransferase activity of NAA40 is required for the expression of *E74*, *E75* and *HR3* genes, we produced a *NAA40* mutant construct by deleting QRKGLG amino acids, located from 148 to 153 region, which constitutes the Acetyl-CoA binding motif of *TcNAA40* (Supplementary Fig. 6). Both wildtype and mutant constructs were transfected into TcA cells, and RNA isolated from these cells was used to quantify *E74*, *E75* and *HR3* mRNA levels. The results demonstrated a significant increase in *E74*, *E75* and *HR3* mRNA levels in TcA cells transfected with wildtype *NAA40* construct, compared to their levels in control cells transfected with either the vector control or *NAA40* mutant (lacking acetyl-CoA binding motif) construct (Fig. 5A). These results demonstrate that acetyltransferase activity of NAA40 is critical for inducing the expression of *E74*, *E75* and *HR3* genes.

### NAA40 localizes into the nucleus

Next, we investigated whether NAA40 has the ability to translocate into the nucleus to acetylate histone proteins linked to the promoters of target genes. To test this hypothesis, we predicted a putative signal peptide sequence (MGRKSSAKSKEKRLKRKEEQ) at the N-terminal region of *NAA40*. Then generated both wildtype *NAA40* (having the predicted signal peptide sequence) and mutant *NAA40* (lacking the predicted signal peptide sequence)-*EGFP* (enhanced green fluorescent protein) fusion constructs. These constructs were transfected into TcA cells, and accumulation of EGFP signal in the nucleus was assessed by confocal imaging. Interestingly, we observed a substantial amount of EGFP accumulation in the nucleus of TcA cells transfected with the wildtype *NAA40* (having the signal peptide)-EGFP fusion construct, compared to the EGFP accumulation in TcA cells transfected with the *NAA40*-EGFP fusion construct lacking the signal peptide which showed more accumulation in the cytoplasm (Fig. 5B and Supplementary Fig. 7). Furthermore, we evaluated whether the lack of a signal peptide could result in the degradation of the NAA40 protein. To investigate this, we extracted proteins from TcA cells at 72 h post-transfections with NAA40 constructs containing or lacking a signal peptide and performed western blotting using the GFP (D5.1) Rabbit monoclonal antibody. Notably, there were no significant differences observed between NAA40 proteins with and without a signal peptide (Supplementary Fig. 7 and Supplementary Data 2). These data suggest that NAA40 localizes into the nucleus to acetylate target histones and may lead to the induction of the target genes.

In addition to acetylating histones, some HATs have been known to directly interact with nuclear hormone receptor complexes or promoters, thereby stimulating the transcription of target genes^12^. Considering this possibility, we determined whether NAA40 directly interacts with the promoters of ecdysone response genes. We predicted the presence of ecdysone response elements (EcREs) within the promoters of *E75* and *HR3* genes. The predicted EcRE regions from these gene promoters were cloned into the pGL3 vector containing the luciferase gene. Then, we tested the ecdysone responsiveness of these constructs by transfecting them into TcA cells and exposing the cells to ecdysone. The luciferase activity significantly increased in cells transfected with the EcRE-luciferase constructs and exposed to ecdysone, compared to those transfected with the empty vector and exposed to DMSO, confirming that these promoter elements are ecdysone responsive (Supplementary Fig. 8). Next, we knocked down *NAA40* in TcA cells and transfected these cells with the EcREs constructs, then assessed the luciferase activity. Surprisingly, no significant difference in the luciferase activity levels was detected between the *NAA40* knockdown and control cells treated with *dsmalE* (Supplementary Fig. 8). These results suggest that NAA40 may not directly interact with the EcREs located in the upstream region of *E75* and *HR3* genes. Though, NAA40 might still induce the expression of ecdysone response genes by acetylating the histone H4 within the nucleus.

### NAA40 regulates ecdysone receptor *EcR-A* gene expression through a positive-feedback loop

**Fig. 6:**
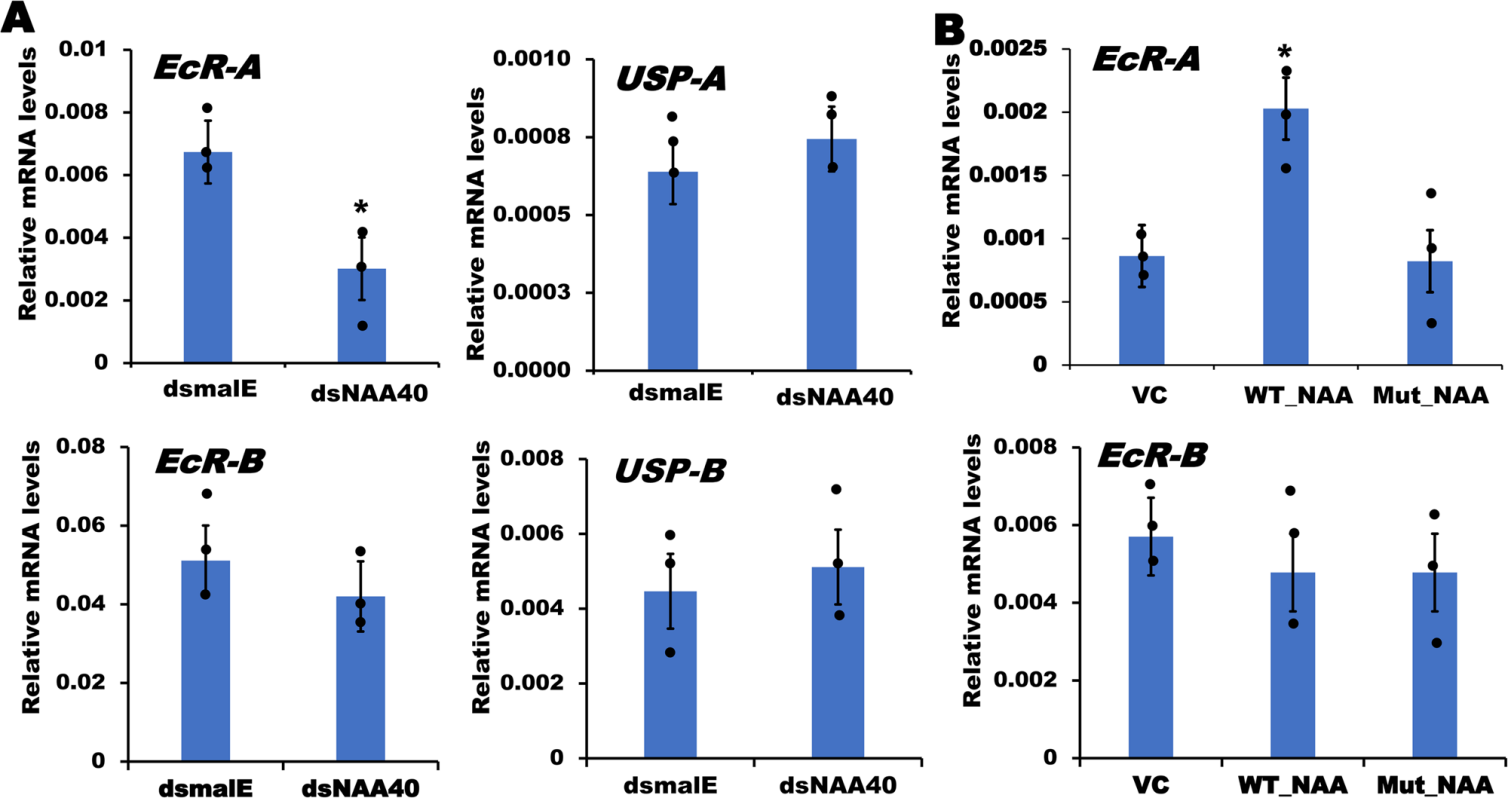
NAA40 regulates ecdysone receptor, *EcR-A* expression. **A** Knockdown of *NAA40* reduced the expression of *EcR-A* but did not affect the expression of *EcR-B* and *USP-A/B*. Asterisk ‘*’ on bars indicates significant differences in the target gene expression levels in cells treated with the *dsNAA40* compared to its expression levels in control cells treated with *dsmalE* at *P* < 0.05. **B** Over-expression of *NAA40* induces the expression of *EcR-A* but not *EcR-B*. The data shows the mean ± SE (*n* = 3). The data were analyzed using student’s *t* test. As*t*erisk ‘*’ on bars indicates significant differences in the target gene expression levels in cells expressing the Wild-type NAA40 (WT_NAA) construct compared to its expression levels in control cells transfected with an empty vector (VC**)** at *P* < 0.05. WT_NAA: Wildtype *NAA40* overexpression, Mut_NAA: Acetyl-CoA binding motif lacking mutant *NAA40* overexpression.

Interestingly, our RNA-seq data revealed a decrease in ecdysone receptor – *EcR* mRNA levels in *NAA40* knockdown *T. castaneum* larvae (Fig. 3C). Similarly, TcA cells also showed reduced expression of *EcR-A* after *NAA40* knockdown (Fig. 6A). These intriguing findings led us to explore the possibility of NAA40 involvement in a positive feedback loop, regulating its own activator, *EcR* expression. To test this hypothesis, we overexpressed *NAA40* in TcA cells and assessed its impact on the expression of genes coding for ecdysone receptors. The mRNA levels of *EcR-A* were significantly enhanced in cells overexpressing the wild-type *NAA40* (Fig. 6B and Supplementary Data 2). However, the acetyl-CoA binding motif lacking mutant *NAA40* overexpression did not increase *EcR-A* expression, indicating that acetyltransferase activity of NAA40 is required for *EcR-A* expression (Fig. 6B). Notably, the expression levels of other ecdysone receptors: *USP-A* and *USP-B* and *EcR-B*, remained unaffected by the knockdown of *NAA40* (Fig. 6A). This result suggests that NAA40 specifically regulates *EcR-A* expression, perhaps through acetylating histones associated with the promoter regions of this gene. To confirm this hypothesis, we performed the ChIP assay using the histone H4 specific Ac-Histone H4 antibody to assess the enrichment of *EcR* promoter in the *NAA40* knockdown samples. Notably, the *EcR* promoter enrichment levels were significantly reduced in the *NAA40* knockdown cells compared to those in cells treated with *dsmalE* (Fig. 4E). These results suggest that NAA40 is involved in the regulation of its own activator, *EcR-A* expression, possibly by increasing the histone H4 acetylation associated with its promoter region.

### NAA40 may act cooperatively with SRC/Taiman to activate ecdysone response genes

**Fig. 7:**
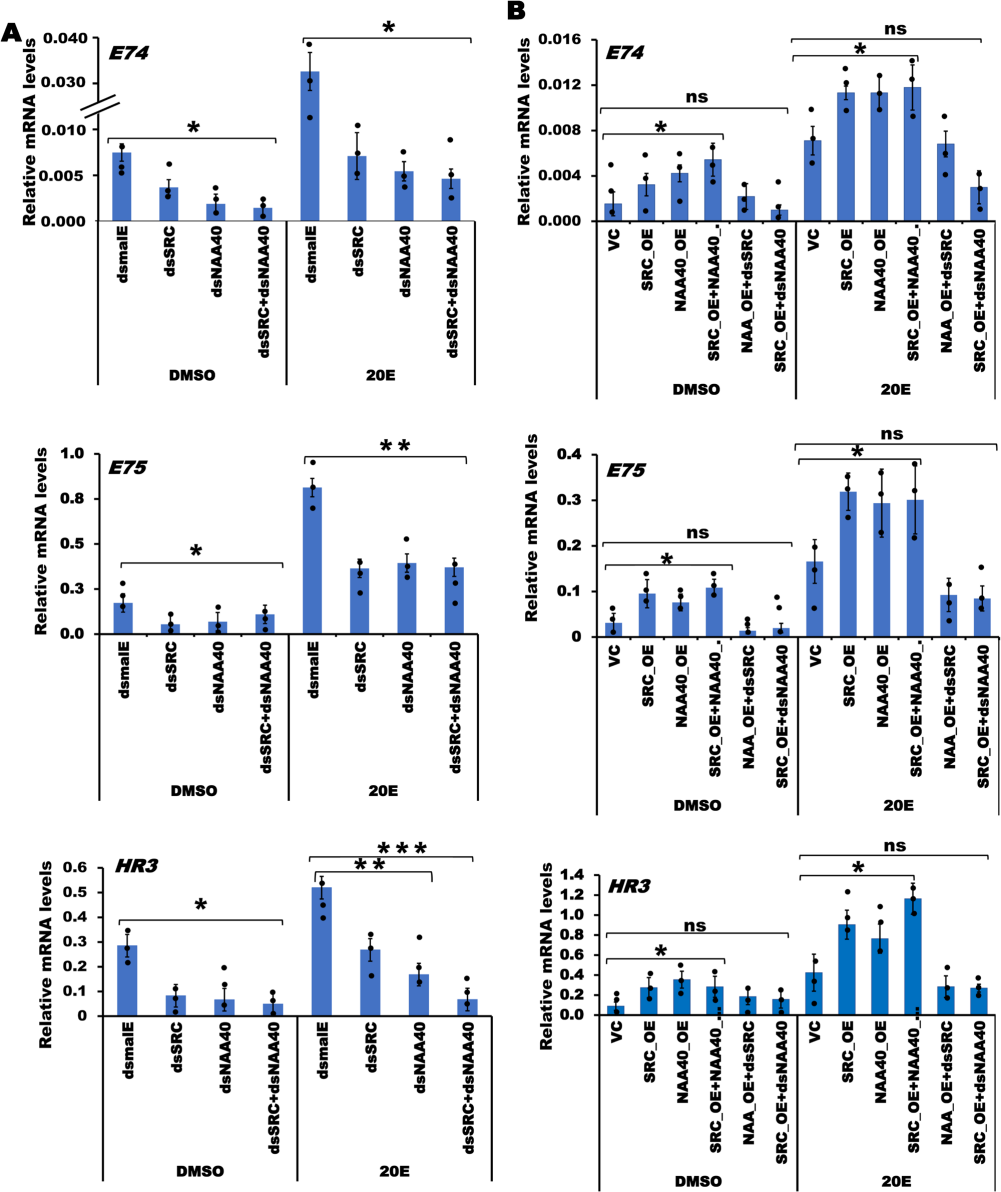
NAA40 and SRC cooperatively regulate the expression of *E74*, *E75* and *HR3* genes. **A** Knockdown of *SRC* or *NAA40* or both together suppressed *E74*, *E75* and *HR3* genes mRNA levels. **B** Overexpression of *NAA40* and SRC induces the expression of *E74*, *E75* and *HR3*. However, in the absence of NAA40, SRC does not increase the above genes expression. The data shows mean ± SE (*n* = 3). Asterisks ‘* or ** or ***’ on bars indicates significant differences in the target gene expression levels in treatments compared to the control treated with *dsmalE* at *P* < 0.05, *P* < 0.01 and *P* < 0.005, respectively analyzed using ANOVA with Post hoc Tukey’s honestly significant difference (HSD) test. Here, ns, not significant, VC: vector control, NAA_OE: Wildtype *NAA40* overexpression, SRC_OE: Steroid receptor co-activator overexpression, SRC_OE + NAA40_OE: Simultaneous overexpression of both *NAA40* and *SRC*, OE: Overexpression, ds: Cognate dsRNA treatment. 20E: 20-hydroxyecdysone, DMSO: Dimethyl sulfoxide.

Previous studies in *D. melanogaster* showed that in the presence of ecdysone, the steroid receptor co-activator (SRC)/taiman interacts with many nuclear receptors, including the ecdysone receptor, to facilitate the activation of ecdysone response genes, especially *E75* and *HR3*^29–32^. In the current study, *NAA40* knockdown led to a reduction in the expression of *E75* and *HR3* genes (Fig. 7A Supplementary Data 2). To understand whether SRC and NAA40 cooperatively regulate these genes expression, we first knocked down *SRC* in TcA cells. Results show a significant decrease in *E74*, *E75* and *HR3* mRNA levels in the *SRC* knockdown cells compared to their levels in the control cells, suggesting that SRC is required for the expression of these genes in TcA cells (Fig. 7A). Next, we examined the effects of simultaneous knockdown of both *SRC* and *NAA40* on the expression of the above genes. Notably, the decrease in mRNA levels of *E74*, *E75* and *HR3* was similar between the cells with simultaneous knockdown of both *SRC* and *NAA40* and cells with knockdown of *NAA40* or *SRC* alone (Fig. 7A). To validate these findings, we transfected TcA cells with expression constructs containing complete open reading frames of wild-type *TcNAA40* and *TcSRC* and exposed these cells to ecdysone or DMSO. Simultaneous overexpression of *SRC* and *NAA40* induced *E74*, *E75* and *HR3* genes. However, the mRNA levels were not significantly different compared to their levels in cells overexpressing *NAA40* or *SRC* alone (Fig. 7B). To verify the cooperation between SRC and NAA40, we knocked down *NAA40* and overexpressed *SRC*. In the absence of *NAA40*, overexpression of *SRC* did not lead to an increase in the expression of ecdysone response genes, suggesting that NAA40 is required for ecdysone/SRC/EcR-mediated induction of *E74*, *E75* and *HR3* genes.

### Arginine methyltransferase 1 (ART1) methylates Arginine 3 (Arg3) in the N-terminal region of histone H4

**Fig. 8:**
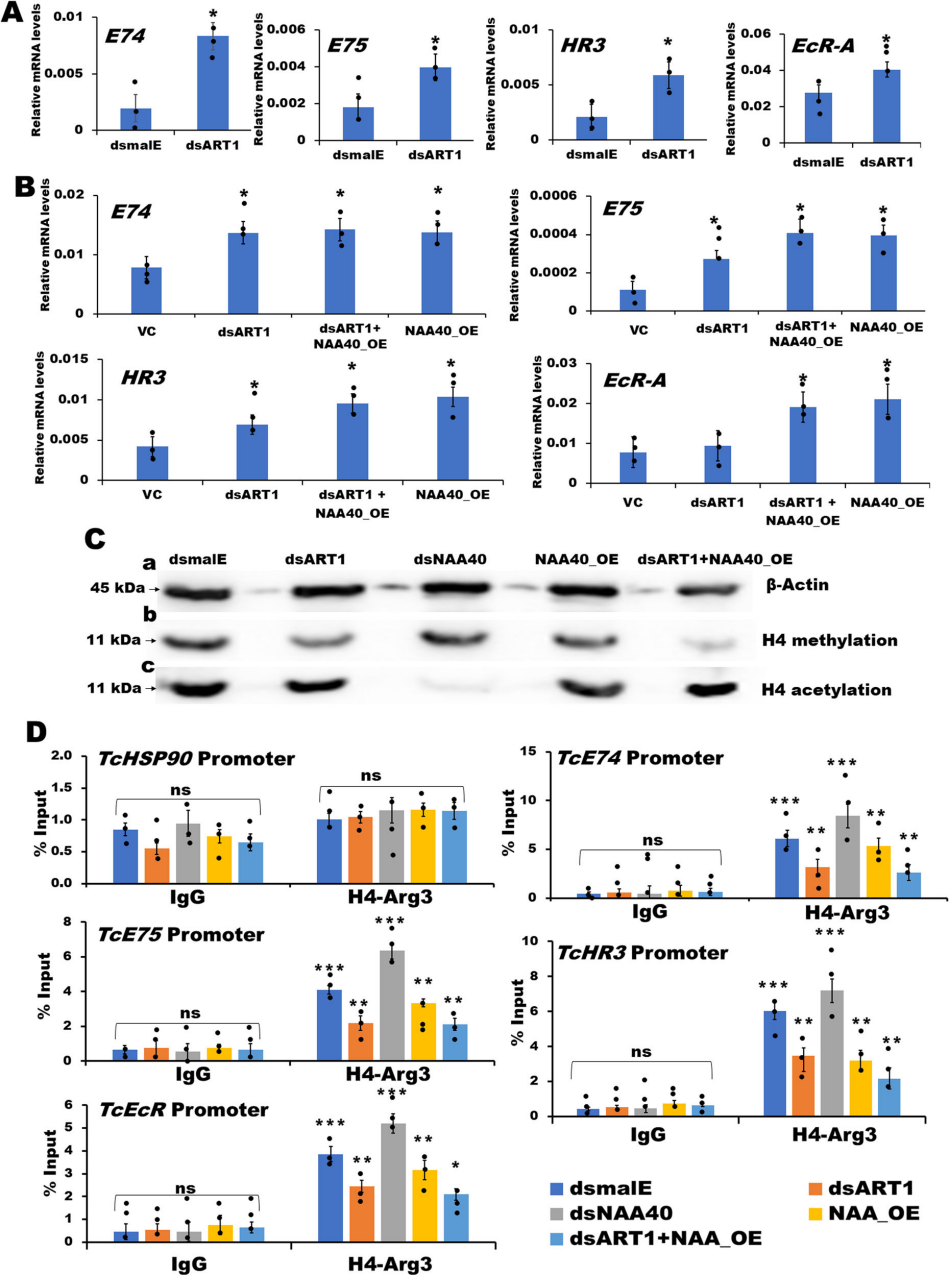
ART1 suppresses ecdysone response genes. **A** Knockdown of *ART1* in *T. castaneum* larvae induces the expression of ecdysone receptor, *EcR-A*, and primary ecdysone response genes, *E74*, *E75* and *HR3* genes. Asterisk ‘*’ on bars indicates significant differences in the target gene expression levels in cells treated with the *dsART1* compared to its expression levels in control cells treated with *dsmalE* at *P* < 0.05. **B** Knockdown of *ART1* in TcA cells or overexpression of *NAA40* induces the expression of *EcR-A*, *E74*, *E75* and *HR3* genes, though there is no additive induction of the above target genes in both *ART1* knockdown and *NAA40* overexpressed cells. The asterisk ‘*’ above the bars indicate significant differences in the expression levels of target gene in various treatments compared to its expression levels in vector control (VC) transfected cells at *P* < 0.05. **C** *ART1* knockdown suppresses histone H4 arginine 3 methylation. The acetylation and methylation levels were determined using nuclear proteins isolated from *NAA40* knockdown, *ART1* knockdown, *NAA40* overexpressed, simultaneous *ART1* knockdown and *NAA40* overexpressed cells. The Ac-Histone H4 and Anti-Histone H4 asymmetric di-methyl Arg3 antibodies were used to detect histone H4 acetylation and methylation, respectively. **a** Loading control protein, β-actin levels in *ART1* knockdown, *NAA40* overexpressed, simultaneous knockdown of *ART1* and *NAA40* overexpressing cells. **b** Histone H4 methylation levels were reduced in *ART1* knockdown, *NAA40* overexpressed and simultaneous knockdown of *ART1* and *NAA40* overexpressed cells. **c** Histone H4 acetylation levels were reduced only in *NAA40* knockdown cells, while *NAA40* overexpression alone or along with *ART1* knockdown increases the H4 acetylation levels. Target protein acetylation/methylation levels among treatments and control were normalized using the loading control protein, β-Actin. **D** Chromatin immunoprecipitation (ChIP) assay showed that *ART1* knockdown significantly reduces the histone H4 methylation levels at the promoters of ecdysone receptor (*EcR*), *E74, E75*, and *HR3* genes in TcA cells. Chromatin was enriched using the Anti-Histone H4 asymmetric di-methyl Arg3 antibody. *HSP90* promoter enrichment levels were used as a control. The rabbit IgG antibody immunoprecipitated chromatin served as a negative control. Each immunoprecipitated sample normalized with its input material and promoter enrichments were represented as percent input. Data are shown as fold enrichment mean ± SE (*n* = 3). The promoter enrichment levels in all groups were compared with those in IgG-enriched *dsmalE* treated control samples. Asterisks ‘* or ** or ***’ on bars indicate significant differences in the enrichment levels of the target gene promoter among different treatments enriched using the H4-Arg3 antibody, in comparison to the those in IgG-enriched *dsmalE* treated control samples, at *P* < 0.05, *P* < 0.01, and *P* < 0.005, respectively. This analysis was conducted using ANOVA with Post hoc Tukey’s honestly significant difference (HSD) test. Here, ‘ns’ denotes not significant.

Interestingly, our RNA-seq data revealed an increase in Arginine methyltransferase 1 (ART1) mRNA levels in *NAA40* knockdown *T. castaneum* larvae (Fig. 3C). Arginine methyltransferase 1 (ART1) methylates Arginine 3 (Arg3) in the N-terminal region of histone H4^33^. Depletion of ART1-mediated methylation of Arg3 leads to an immediate acetylation of histone H4 and increase in the expression of target genes^33^. To test the potential involvement of ART1 in the regulation of primary ecdysone response genes, we knocked down *ART1* in both *T. castaneum* larvae and TcA cells. We observed an increase in the expression of *EcR-A*, *E74*, *E75*, and *HR3* in both *T. castaneum* larvae and TcA cells treated with *dsART1*, compared to their levels in respective controls treated with *dsmalE* (Fig. 8A, B and Supplementary Data 2). To further explore the intricate interplay between ART1 and NAA40, we knockdown *ART1* and overexpressed *NAA40* in TcA cells. Results show an increase in the expression levels of *EcR-A*, *E74*, *E75* and *HR3* genes (Fig. 8B).

To determine whether ART1-mediated methylation has a role in the suppression of these genes, *ART1* was knocked down in TcA cells. Nuclear proteins were extracted from these cells and performed western blotting using the Anti-Histone H4 asymmetric di-methyl Arg3 antibody to detect histone H4-Arg3 methylation levels. The histone H4 methylation levels decreased in the *ART1* knockdown cells compared to the control cells treated with *dsmalE*. However, *NAA40* knockdown did not significantly alter the histone H4 methylation levels (Fig. 8C and Supplementary Figs. 5 and 12). Conversely, overexpression of *NAA40* led to slight reduction in the histone H4 methylation levels. While the knockdown of *ART1* and overexpression of *NAA40* further reduced the histone H4 methylation levels.

To explore whether ART1 methylated histone H4 is associated with *EcR*, *E74*, *E75* and *HR3* promoters, we knockdown *ART1* in TcA cells and performed ChIP assays. Chromatin from treated cells enriched using the Anti-Histone H4 asymmetric di-methyl Arg3 antibody. The enrichment levels of *EcR*, *E74*, *E75* and *HR3* promoters decreased in the *ART1* knockdown cells compared to control cells treated with *dsmalE* (Fig. 8D). Interestingly, overexpression of *NAA40* resulted in reduced enrichment levels of the *EcR*, *E74*, *E75* and *HR3* promoters in the chromatin enriched using the Anti-Histone H4 asymmetric di-methyl Arg3 antibody (Fig. 8D). These results indicate that ART1 methylated histone H4-Arg3 mark is possibly associated with the promoters of *EcR*, *E74*, *E75* and *HR3* genes and potentially plays a role in ART1-mediated suppression of these genes by methylating histone H4.

## Discussion

Epigenetic modifiers play a crucial role in regulating gene expression during insect growth, development, and metamorphosis^34^. This study focuses on NAA40, a histone acetyltransferase, and its involvement in ecdysone signaling during metamorphosis of *T. castaneum*. Through RNAi-mediated knockdown of various HATs, including *NAA40*, we identified key HATs that are essential for larval growth, development, and metamorphosis of *T. castaneum*. Among the HATs studied, *NAA40* knockdown resulted in severe phenotypes such as larval growth arrest, blocked larval-pupal metamorphosis, and death at the prepupal stage, which are similar to *EcR* knockdown effects in insects^35^. Based on these observations, we hypothesized that NAA40 may modulate ecdysone regulation of growth, development, and metamorphosis of *T. castaneum*. Additionally, *NAA40* developmental expression levels correlate with ecdysteroid titers reported previously^24^. Studies in TcA cells verified this hypothesis, ecdysone through its receptor EcR, induces *NAA40* expression. In contrast, histone deacetylases (HDACs) are suppressed by the anti-metamorphic hormone – juvenile hormone (JH) to prevent precocious metamorphosis of *T. castaneum* larvae^13–15^. Notably, ecdysone does not influence the expression of *HDACs*, and JH does not affect *NAA40* expression. These findings highlight the distinct roles of different epigenetic modifiers in JH and ecdysone action in regulation of gene expression during metamorphosis.

Interestingly, *NAA40* knockdown resulted in decreased expression of its own activator, *EcR-A*, while *NAA40* overexpression increased *EcR-A* mRNA levels, suggesting a positive feedback loop. Positive feedback loops are crucial for amplifying signaling and achieving desired phenotypes^36^. In insects, ecdysone plays a key role in the larval-pupal developmental transition that relies on higher and prolonged ecdysone signaling during metamorphosis. Our studies showed that NAA40 is perhaps involved in amplifying ecdysone signaling during *T. castaneum* metamorphosis. During metamorphosis, increased ecdysteroid titers facilitate the dimerization of EcR/USP receptor complex, that may bind to predicted EcRE elements (GGTTTGATGATCC) in the *NAA40* promoter, thereby inducing its expression. However, further investigations are necessary to confirm this interaction in the upstream region of NAA40. Interestingly, NAA40 further enhances *EcR* expression to amplify ecdysone signaling to ensure a successful larval-pupal transition. Notably, knockdown of *NAA40* prevented a successful larval-pupal transition in *T. castaneum*, indicating that NAA40-mediated positive feedback regulation may play a critical role in amplifying ecdysone signaling during metamorphosis.

Ecdysone triggers the activation of various transcription factors, including E74, E75, and HR3, to facilitate the transition from larval to pupal stages, with the help of NAA40. Previous studies demonstrated that the ecdysone receptor complex recruit specific histone acetyltransferases (HATs) such as CBP, SRC, and nucleosome remodeling factor (NURF). And these complexes interact with ecdysone response elements (EcREs) present in the promoters of ecdysone response genes, activating their expression^37–39^. Unlike other HATs, NAA40 lacks a DNA binding domain but contains a conserved acetyl-CoA binding motif, QRKGLG, which perhaps responsible for transferring acetyl groups from acetyl-CoA to histones for N-terminal acetylation of histone H4 (Supplementary Fig. 6). Experiments involving transfection of *NAA40* mutant construct (lacking the above acetyl-CoA binding motif) failed to support ecdysone induction of its response genes. Additionally, transfection of TcA cells with EcREs of *E75* and *HR3* gene promoter constructs and knockdown of *NAA40*, did not exhibit any effect on ecdysone-induced luciferase activity. This data suggests that NAA40 does not directly interact with the promoters of *E75* and *HR3* genes. Instead, NAA40 likely regulates the expression of ecdysone response genes by modulating the acetylation levels of histone H4 localized in their promoters.

NAA40 (NatD) distinguishes itself from other NATs as it independently functions without co-activators or auxiliary subunits^2,40^. A previous study in *D. melanogaster*, the steroid receptor co-activator (SRC), a HAT, was found to be essential for the expression of *E75* and *HR3* genes^30^. Our data in *T. castaneum* revealed that NAA40 is also required for the expression of *E75* and *HR3* genes. Knockdown and overexpression experiments demonstrated that SRC alone could not support the expression of these genes in the absence of NAA40, suggesting that both SRC and NAA40 are required for the expression of ecdysone response genes. These results are similar to previous study showed how SRC and CBP synergize to regulate estrogen receptor expression^41^. SRC and CBP act as co-activators, interacting with ecdysone receptor complex in the presence of ecdysone, then bind to EcREs in the promoters of ecdysone response genes, and inducing target gene expression^37^. However, our studies indicated that the luciferase gene regulated by EcREs from *E75* and *HR3* promoters was unaffected by *NAA40* knockdown. This suggests that NAA40 may not directly interact with the promoters to activate their expression. Instead, NAA40 may acetylate the histone H4 mark associated with the *E75* and *HR3* gene promoters. This acetylation could potentially facilitate the recruitment of ecdysone receptor complexes and co-activators such as SRC and CBP to the promoters of ecdysone-response genes, ultimately leading to their activation. However, additional investigations are necessary to validate this hypothesis further.

Another intriguing finding of this study is the contrasting actions of ART1-mediated histone H4 methylation, which suppresses ecdysone activity. Histone modifications can yield different effects on the same target, depending on the specific modifier involved. For instance, histone H3 serine 10 phosphorylation stimulates histone H3 lysine 14 acetylation^42^. Similarly, knockdown of *ART1* leads to increased histone H4 acetylation due to the depletion of histone H4 arginine 3 methylation in erythroid cells^43^. In Hela cells, ART1-mediated methylation of histone H4 arginine 3 facilitates subsequent acetylation of histone H4 tails by acetyltransferases (HATs)^43^. Conversely, histone H4 acetylation prevents its methylation by ART1^33^. Our data and other studies suggest that histone H4 acetylation by NAA40 may potentially influence histone H4 arginine 3 methylation mediated by ART1. Additionally, the developmental expression patterns of *ART1* and *NAA40* during the penultimate and last instar larval and pupal stages suggest that histone H4 methylation may be replaced by acetylation upon *NAA40* expression in response to increased ecdysteroid levels (Supplementary Fig. 9). This shift occurs before metamorphosis initiation, leading to the activation of ecdysone-induced transcription factors. In *D. melanogaster*, ART1 acts as a repressor of the ecdysone receptor, EcR, by methylating histone H4 associated with the *EcR* promoter^44^. Similarly, our findings suggest that ART1-mediated methylation of arginine 3 on histone H4 may be localized at the *EcR* promoter, potentially leading to the repression of *EcR* and other ecdysone response genes. These data suggest that the regulation of histone H4 methylation and acetylation levels plays a vital role in modulating the ecdysone response. ART1 may function as an EcR repressor, while NAA40-induced acetylation could counteract ART1 repressive activity, thereby promoting *T. castaneum* metamorphosis.

In conclusion, our study highlights the role of NAA40 in *T. castaneum* metamorphosis. Ecdysone triggers significant changes in gene expression through chromatin modifications mediated by epigenetic modifiers^45^. The *NAA40* gene, induced by ecdysone, likely plays a crucial role in these chromatin modifications, involved in ecdysone regulation during metamorphosis. Based on our findings, we propose a model for the epigenetic modulation of ecdysone action during metamorphosis (Supplementary Fig. 10). Ecdysone induces the *NAA40* gene expression, and subsequently, NAA40 acetylates histone H4, possibly localized at the promoters of key ecdysone response genes coding for *EcR, E74, E75, and HR3*. This acetylation facilitates an increase in their expression and promotes larval-pupal metamorphosis. On the other hand, in the absence of ecdysone, ART1 methylates histone H4 associated with the promoters of *EcR*, *E74*, *E75*, and *HR3* genes, repressing their expression and inhibiting metamorphosis. The dynamic changes in histone acetylation and methylation levels at the promoters of ecdysone response genes play pivotal roles in modulating ecdysone action and regulating insect metamorphosis. Our findings shed light on the intricate epigenetic regulation involved in ecdysone-induced metamorphosis in insects.

## Methods

### Insect strains

The North American Georgia strain GA-1^46^ of the red flour beetle *T. castaneum* (Herbst) was used in experiments. The beetles were reared as described previously^13^.

### Cell culture

*Tribolium castaneum* cell line, BCIRL-TcA-CLG1 (TcA) established from a co-culturing adult, and pupal tissues were obtained from Dr. Goodman^47^. These cells were maintained in EX-CELL 420 medium (Sigma-Aldrich, St. Louis, MO) supplemented with 10% FBS (Fetal Bovine Serum) (VWR Seradigm Fetal Bovine Serum, Radnor, PA) and 1 µg/ml of Penicillin-Streptomycin antibiotic mix in 5 ml sterile flasks at 28°C.

### Hormone treatments

Technical grade 20-Hydroxyecdysone (20E, Catalog No: H5142, Sigma-Aldrich, St. Louis, MO) and juvenile hormone III, (JH III, Catalog No: J2000, Sigma-Aldrich, St. Louis, MO) were dissolved in dimethyl sulfoxide (DMSO) (Sigma-Aldrich, St. Louis, MO). Pre-seeded cells were exposed to 10 μM of 20E or JH III for 6 h. The control cells were treated with the same volume of DMSO.

### Gene expression analysis

Total RNA was extracted from treated and control insects or cells using the TRI reagent-RT (Catalog No: RT 111, Molecular Research Center Inc., Cincinnati, OH). Complementary DNA (cDNA) was synthesized from 2 μg of total RNA samples using the M-MLV reverse transcriptase (Catalog No: 28-025-013, Invitrogen, USA) in a 20 μl reaction as per the manufacturer’s instruction. The expression of the target genes was assessed using the iTaq Universal SYBR Green Supermix by following the manufacturer’s recommendations (Catalog No: 1725120, Bio-Rad, Hercules, CA). The relative mRNA levels were calculated as described previously^48^ after normalizing with a reference gene, Ribosomal protein 49 (*RP49*).

### Knockdown of target genes

Gene fragments ranging from 300 to 500 bp were amplified using gene-specific primers (Supplementary Table 5). These primers were designed to incorporate T7 promoter sequences at the 5’ end. cDNA was used as a template for amplifying target genes. Double-stranded RNA (dsRNA) was synthesized using the MEGAscript T7 kit (Catalog No: A57622, Invitrogen, USA) and the PCR product was purified following the manufacturer’s instructions. Newly molted last instar larvae were microinjected with 1 μg of cognate dsRNA. While control larvae were injected with *dsmalE*: dsRNA targets the gene coding for a maltose-binding protein of *E. coli*.

### RNA-sequencing and analysis

In our previous publications, we have provided a detailed description of the method followed in this study^9,49^. Briefly, RNA-seq libraries were prepared using 2 µg of total RNA per replicate. These libraries were size selected, pooled and sequenced using the Illumina Hiseq 4000 sequencer at Duke University Sequencing and Genomic Technologies (NC, USA). Raw reads after quality control – demultiplexed, trimmed, and were mapped back to the *T. castaneum* reference genome (assembly Tcas5.2). This mapping was performed using the CLC genomic workbench pipeline (Version 11.0.1, Qiagen Bioinformatics, Valencia, CA) with pre-optimized parameters, such as unique exon mapping, mismatch cost = 2, insertion cost = 3, deletion cost = 3, length fraction = 0.8, similarity fraction = 0.8. Differential gene expression analysis was performed using the “Empirical analysis of DGE” (EDGE) tool within the CLC genomic workbench with uniquely mapped reads. Transcripts exhibiting a fold change of ≥ 2 and a false discovery rate (FDR) corrected *P*-value cutoff of ≤ 0.05 were considered as differentially expressed genes. The K-mer clustering tool in the CLC genomics workbench was utilized to group transcripts. Functional annotation of the selected transcripts was performed using the Blast2Go pro plugin within the CLC genomics workbench. Gene ontology (GO) enrichment analysis was done using the Web Gene Ontology Annotation Plot (WEGO), by plotting of the GO information of the differentially expressed genes against the GO terms of *T. castaneum* genome^50^. The GO terms of *NAA40* knockdown RNA-seq data compared with the GO terms of the *EcR* knockdown data from *Drosophila*^25^ and generated a Venn diagram to represent the common and unique GO terms enriched between the two datasets.

### Western blot analysis and Chromatin immunoprecipitation (ChIP) assay

Chromatin-bound nuclear proteins were extracted and performed Western blotting by following previously established protocols^9,12,13,51^. Briefly, treated cells or larval samples were lysed using a lysis buffer containing 50 mM tris (pH 7.5), 150 mM NaCl, 1 mM EDTA, 1% NP-40 and 0.1% cOmplete Mini Protease Inhibitor Cocktail (Cat No: C852A34, Roche Diagnostics, USA) on ice. Following a 10 min incubation on ice, 1/10 volume of 5 M NaCl was added to release chromatin-bound proteins. Then nuclear proteins were precipitated by adding 2 volumes of ice-cold acetone and allowed to precipitate overnight at −20 °C, followed by centrifugation at 3000 g for 15 min at 4 °C. The pellet was washed with ice-cold acetone and dissolved in 1% Sodium dodecyl sulfate (SDS). A previously optimized concentration of 50 μg of total protein extracted from treated and control TcA cells or larvae were used for western blotting. Histone H4 acetylation modifications were detected using 1:1000 diluted Ac-Histone H4 (E-5) mouse monoclonal antibody (mAb) (Catalog No: sc-377520, Santa Cruz Biotechnology, Inc. USA). This antibody detects Ser1, Lys5, Lys8, and Lys12 acetylated residues in the histone H4 tail. The acetylation of individual amino acids, Lys5, Lys8 and Lys12 in the histone H4 tail were detected using the Acetyl-Histone H4 antibody sampler kit that includes Acetyl-Histone H4 (Lys5) mAb, Acetyl-Histone H4 (Lys8) polyclonal antibody (pAb), Acetyl-Histone H4 (Lys12) mAb and Histone H4 mAb (Catalog No: 8346, Cell Signaling Technology, USA). Histone H4-Arg3 methylation levels were determined using the 1:1000 diluted Anti-Histone H4 asymmetric di-methyl Arg3 antibody which detects methylation of Arginine 3 of histone H4 (Catalog No. ab194683, Abcam, USA). The GFP (D5.1) Rabbit mAb (Catalog No: 2956, Cell signaling Technology, USA) was employed to detect signal peptide containing wildtype NAA40 protein and signal peptide lacking NAA40 protein. Mouse IgG kappa binding protein (m-IgG_k_ BP) (Catalog No: sc-516102, Santa Cruz Biotechnology, Inc. USA) conjugated to Horseradish Peroxidase (HRP) or Anti-rabbit IgG-HRP-linked Antibody (Catalog No: 7074, Cell Signaling Technology, USA) were used as secondary antibodies. The blots were developed by incubating them in a chemiluminescence reagent, Supersignal^TM^ West Femto Maximum Sensitivity Substrate (Catalog No: PI34095, ThermoFisher, USA). Band densities were quantified using Image-J software. The mean band intensity of target protein acetylation/methylation among treatments and control was normalized using the loading control protein, β-Actin. Subsequently, normalized protein acetylation/methylation levels were then represented as relative fold change compared to the control.

ChIP assays were performed following previously described protocol^12^. Briefly, *NAA40* was knocked down in TcA cells for 72 h. Then cells were fixed using 1% formaldehyde to cross-link DNA and associated proteins. The fixed cells were harvested, and the cross-linked chromatin was immunoprecipitated using the 5 μg of Ac-Histone H4 (E-5) or Anti-Histone H4 asymmetric di-methyl Arg3 antibody or IgG (negative control) antibodies by following the manufacturer protocol. The DNA recovered from the immunoprecipitated samples was used for promoter enrichment analysis using qPCR. Each immunoprecipitated sample was normalized with its input material and promoter enrichments were represented as a percent input.

### Generation of plasmid constructs and transfection into TcA cells

Polyubiquitin promoter was PCR amplified (Supplementary Table 5) from *T. castaneum* genomic DNA and cloned into the pIEx-4 vector digested with *Nhe* I and *Nco* I restriction enzymes. This vector is designated as the PolyUbi-pIEx-4 vector. Next, PCR amplified the full-length coding sequence of *T. castaneum NAA40* gene and cloned it into the PolyUbi-pIEx-4 vector using *Nco* I and *Hind* III restriction enzymes. Created a mutant form of *TcNAA40* lacking acetyl-CoA binding motif (QRKGLG amino acids – essential for NAA40 acetyltransferase activity) by performing site-directed mutagenesis (Cat No: E0554S, New England Biolabs, USA). To study the cooperativity between NAA40 and SRC, we used the TcSRC construct developed in our previous study^31^. For overexpression of recombinant proteins, TcA cells were transfected with 500 ng of respective plasmid constructs using the X-tremeGENE^™^HP DNA transfection reagent (Catalog No: 6366244001, Roche Diagnostics, USA). After 72 h, the total RNA was extracted from the transfected cells and used to assess the expression levels of target genes.

Using the *B. mori* ecdysone response elements (*EcREs*) consensus sequences, we predicted the presence of ecdysone response elements (*EcREs*) in the upstream region of *TcE75* and *TcHR3* genes^52^. The predicted *EcREs* were cloned into the pGL3 vector, which contains the luciferase gene, to drive luciferase gene expression. We tested the ecdysone response of these constructs by transfecting them into TcA cells and exposing the cells to either 10 μM of 20E or DMSO. In the next experiment, we treated TcA cells with *dsNAA40 or dsmalE* and then transfected these cells with *EcRE* constructs. After 48 h, the cells were exposed to 10 μM of 20E or DMSO for an additional 24 h and assessed the luciferase activity. To study the localization of exogenously expressed *NAA40*, we predicted a putative signal peptide sequence at the N-terminal region of *NAA40*. The complete ORF of *NAA40* and *NAA40* lacking signal peptide sequence were PCR amplified (Supplementary Table 5) and then fused with the EGFP complete ORF sequence and cloned into the pIEx-4 vector containing the polyubiquitin promoter using the Gibson assembly kit, following the manufacturer’s protocol (Catalog No: A46627, ThermoFisher, USA). TcA cells were transfected with the above (*EGFP* + *NAA40* with signal peptide or *EGFP* + *NAA40* lacking signal peptide) fusion constructs. After 72 h post-transfection, the cells were washed with 1X phosphate buffer saline (PBS), fixed, and mounted using the Everbrite mounting medium containing DAPI (Sigma-Aldrich, St. Louis, MO). Cells were imaged using a Leica TCS SP8 DLS (Digital LightSheet) confocal microscope at 63X magnification. The fluorescence intensities of DAPI and EGFP were quantified using Image J software and normalized against DAPI, following a method described previously^53^. The mean relative fluorescence units (RFU) represented as a relative fold change.

### Statistics and reproducibility

The mortality data significance between treatments and control was analyzed using one-way ANOVA with the GraphPad Prism v5.0 software. For multi-group comparisons, ANOVA with Post hoc Tukey’s honestly significant difference (HSD) test was employed. Student’s *t* test or One-way ANOVA was used to analyze the expression data between treatments and control. Statistical significance was defined as *p < 0.05, **p < 0.01, ***p < 0.001; ns, not significant. All the experiments were conducted with a minimum of three repetitions, each consisting of four biological replicates.

### Supplementary information

Supplementary information

Description of additional supplementary files

Supplementary data 1

Supplementary data 2

## Data Availability

The RNA-seq data reported in this study have been deposited into the National Center for Biotechnology Information’s Sequence Read Archive (NCBI-SRA) database with the following accession number: PRJNA612693. All data supporting the findings of the paper are present in the paper/Supplementary information/Data 1. Raw data used for plots are available as Supplementary Data 2.
