# Supplementary material for: N(alpha)-acetyltransferase 40-mediated histone acetylation plays an important role in ecdysone regulation of metamorphosis in the red flour beetle, Tribolium castaneum
Source: Commun Biol. 2024 May 3;7:521. doi: 10.1038/s42003-024-06212-7 (PMC11068786; doi:10.1038/s42003-024-06212-7)
Supplement: Supplementary file 1 — Supplementary information [file 42003_2024_6212_MOESM1_ESM.docx]

Supplementary information

**N(alpha)-acetyltransferase 40-mediated histone acetylation plays an important role in ecdysone regulation of metamorphosis in the red flour beetle, *Tribolium castaneum***

Sharath Chandra Gaddelapati^1,2^, Smitha George^1^, Anilkumar Moola^1^, Karthi Sengodan^1^ and Subba Reddy Palli^1^*

^1^Department of Entomology, College of Agriculture, Food and Environment, University of Kentucky, Lexington, Kentucky 40546, USA

^2^Current Affiliation: Donald Danforth Plant Science Center, St. Louis, Missouri 63132, USA

*Corresponding author

Phone: 859-257-4962

Fax: 859-323-1120

Email: [rpalli@email.uky.edu](mailto:rpalli@email.uky.edu)

Supplementary Table 1. Gene group: Lysine acetyltransferases (KATs) in *T. castaneum*

| **HGNC gene symbol** | **Gene name** | ***Tribolium* gene symbol and locus tag** | ***Drosophila* gene symbol** |
| --- | --- | --- | --- |
| KAT5 | Histone acetyltransferase Tip60 | LOC664310-TC008142 | Tip60 |
| KAT6A | Histone acetyltransferase KAT6A | LOC659405-TC006477 | Enok |
| KAT7 | Histone acetyltransferase KAT7 | LOC658964-TC011945 | Chm |
| KAT8 | Histone acetyltransferase KAT8 | LOC656837-TC003573 | Mof |
| ATAT1 | Alpha-tubulin N-acetyltransferase 1 | LOC660866-TC003198 | Tat |
| ESCO1/2 | N-acetyltransferase ESCO2 | LOC661939-TC009742 | Eco |
| HAT1 | Histone acetyltransferase type B catalytic subunit | LOC655088-TC002077 | Hat1 |
| KAT2A | Histone acetyltransferase KAT2A | LOC658128-TC014774 | Gcn5 |
| TAF1 | Transcription initiation factor TFIID subunit 1 | LOC658493-TC002507 | Taf1 |
| ELP3 | Elongator complex protein 3 | LOC656399-TC004103 | Elp3 |
| KAT14 | Cysteine-rich protein 2-binding protein | LOC103313557-TC009267 | Atac2 |
| MCM3AP | Protein x-mas-2 | LOC657257-TC008518 | Xmas |

Supplementary Table 2. Gene group: N-terminal acetyltransferases (NATs) in *T. castaneum*

| **HGNC gene symbol** | **Gene name** | ***Tribolium* gene symbol and locus tag** | ***Drosophila* gene symbol** |
| --- | --- | --- | --- |
| GNPNAT1 | Probable glucosamine 6-phosphate N-acetyltransferase | LOC660783-TC009619 | Gnpnat |
| NAA10 | N-alpha-acetyltransferase 10 | LOC658076-TC000030 | Vnc |
| NAA20 | N-alpha-acetyltransferase 20 | LOC664281-TC007251 | Naa20A |
| NAA30 | N-alpha-acetyltransferase 30 | LOC664218-TC008129 | Naa30A |
| NAA40 | N-alpha-acetyltransferase 40 | LOC655482-TC015921 | Naa40 |
| NAA50 | Probable N-acetyltransferase san | LOC107397753-TC034643 | San |
| NAA60 | N-alpha-acetyltransferase 60 | LOC660507-TC010378 | Naa60 |
| NAA80 | N-acetyltransferase 6 | LOC103313702-TC008846 | Naa80 |
| NAT9 | N-acetyltransferase 9-like protein | LOC659678-TC000126 | CG11539 |
| NAT10 | RNA cytidine acetyltransferase | LOC662819-TC007435 | L(1)G0020 |
| SATL1 | Diamine acetyltransferase 2 | LOC662606-TC013490 | CG4210 |

Supplementary Table 3. Gene group: N(alpha)-acetyltransferase subunits (NAAs) in *T. castaneum*

| **HGNC gene symbol** | **Gene name** | ***T. castaneum* gene locus tag** |
| --- | --- | --- |
| NAA16 | N(alpha)-acetyltransferase 16, NatA auxiliary subunit | LOC664508-TC008190 |
| NAA25 | Phagocyte signaling-impaired protein, NatB auxiliary subunit | LOC100141721-TC015420 |
| NAA35 | N(alpha)-acetyltransferase 35, NatC auxiliary subunit | LOC655496-TC004679 |
| NAA38 | N(alpha)-acetyltransferase 38, NatC auxiliary subunit | LOC664214-TC008128 |

**Supplementary Table 4**. **Knockdown of *TcNAA40* or *Drosophila* ecdysone receptor (*EcR*) affects some common gene ontologies.**

| **GO ID** | **GO Term** | **Gene Name** |
| --- | --- | --- |
| GO:0040003 | Chitin-based cuticle development | Larval cuticle protein A2B |
| GO:0006094 | Gluconeogenesis | Glycerol-3-phosphate dehydrogenase [NAD(+)], cytoplasmic |
| GO:0006030 | Chitin metabolic process | Probable chitinase 3 |
| GO:0010025 | Wax biosynthetic process | Fatty acyl-CoA reductase 1-like |
| GO:0007218 | Neuropeptide signaling pathway | Neurotactin |
| GO:0006805 | Xenobiotic metabolic process | Carboxylesterase-6 |
| GO:0043401 | Steroid Hormone Mediated Signaling Pathway | Hormone receptor 3 |
| GO:0009267 | Cellular response to starvation | GTPase-activating protein CdGAPr |
| GO:0002028 | Regulation of sodium ion transport | Sodium/hydrogen exchanger 11-like |
| GO:0040003 | Chitin-based cuticle development | Cuticle protein 19 |
| GO:0006364 | rRNA processing | Nuclear polyadenylated RNA-binding protein 3 |
| GO:0006869 | Lipid transport | Apolipophorin-III |
| GO:0055085 | Transmembrane transport | ATP-binding cassette sub-family A member 3-like |
| GO:0006508 | Proteolysis | Serine protease P80 |
| GO:0043401 | Steroid Hormone Mediated Signaling Pathway | Ecdysone-inducible protein E75 |

***Note***: A comparison of GO terms between *TcNAA40* knockdown RNA-seq data and *Drosophila* ecdysone receptor (EcR) knockdown RNA-seq data revealed that both *TcNAA40* and *DmEcR* knockdown affects some common gene ontologies listed in the above table.

Supplementary Table 5. List of primers used in this study.

| **Gene/Primer name** | **Primer Sequence (5' -> 3')** |
| --- | --- |
| **dsRNA synthesis** | |
| dsTC008142F | TAA TAC GAC TCA CTA TAG GGTTGCCTCAAGTACCGAAAGAG |
| dsTC008142R | TAA TAC GAC TCA CTA TAG GGCTTGTGCCCAGTAACTCCTATAC |
| dsTC006477F | TAA TAC GAC TCA CTA TAG GGGACTCCACCTCCTGAAACTAAA |
| dsTC006477R | TAA TAC GAC TCA CTA TAG GGCCGCCGCACTCATACTAAA |
| dsTC011945F | TAA TAC GAC TCA CTA TAG GGGGAAGGAACCACCGAAGAAG |
| dsTC011945R | TAA TAC GAC TCA CTA TAG GGCTGACCACGGCCTTAGATTTAG |
| dsTC003573F | TAA TAC GAC TCA CTA TAG GGCACCAGGAATCAGAAGAGAAGG |
| dsTC003573R | TAA TAC GAC TCA CTA TAG GGTTGCTCTGTCGGCCATATTC |
| dsTC003198F | TAA TAC GAC TCA CTA TAG CATCGACCGACCAAGTGAAA |
| dsTC003198R | TAA TAC GAC TCA CTA TAG TGCTGCTGCTGGTAGTATTG |
| dsTC009742F | TAA TAC GAC TCA CTA TAG GGCGACCTACGCAACGGTAAA |
| dsTC009742R | TAA TAC GAC TCA CTA TAG GGGCGCCAAATACGAAGTTTCTC |
| dsTC002077F | TAA TAC GAC TCA CTA TAG GACATGGCCCACCAGATATT |
| ds TC002077R | TAA TAC GAC TCA CTA TAG GTCCTCCAGGTCTATGAAGTTG |
| dsTC014774F | TAA TAC GAC TCA CTA TAG GGCCAATCGGCGGTATTTGTTTC |
| dsTC014774R | TAA TAC GAC TCA CTA TAG GGGACTGAAGTGAACTCGGTGTAG |
| dsTC002507F | TAA TAC GAC TCA CTA TAG CAATGCTGCCGTCCAAATAC |
| dsTC002507R | TAA TAC GAC TCA CTA TAG TTGGGTGACCATGAGGAAAG |
| dsTC004103F | TAA TAC GAC TCA CTA TAG GGACGTCAATGAGGGCCATAAG |
| dsTC004103R | TAA TAC GAC TCA CTA TAG GGTAATCCGGCCTTGTCTCAATC |
| dsTC009267F | TAA TAC GAC TCA CTA TAG CGAGTCGTTTGTTAGGGAGAAA |
| dsTC009267R | TAA TAC GAC TCA CTA TAG CGTTCGTGAGAGCAGCATATAA |
| dsTC008518F | TAA TAC GAC TCA CTA TAG CATCACTTCCCGTCTCATACTT |
| dsTC008518R | TAA TAC GAC TCA CTA TAG GTGACGCTAGTTGGGTTATCT |
| dsTC009619F | TAA TAC GAC TCA CTA TAG GGGCAGTCTGGTGGTTACTACATC |
| dsTC009619R | TAA TAC GAC TCA CTA TAG GGACGGAATAAGCGGGTCTTTAC |
| dsTC000030F | TAA TAC GAC TCA CTA TAG GGCGAAGATGGAAGAGGACAATGAG |
| dsTC000030R | TAA TAC GAC TCA CTA TAG GGCATCTTCGCCATCTGCGTAATA |
| dsTC007251F | TAA TAC GAC TCA CTA TAG GGGACATGTGACAGCCCTAACT |
| dsTC007251R | TAA TAC GAC TCA CTA TAG GGGGTCGCCTGAGTAATATTCCAA |
| dsTC008129F | TAA TAC GAC TCA CTA TAG GGCGAAGACGAGGGCATTAACA |
| dsTC008129R | TAA TAC GAC TCA CTA TAG GGACCGGTAGGTGTAGATGGAATA |
| dsTC015921F | TAA TAC GAC TCA CTA TAG GGAGTTGGGATAGGCACCATAAC |
| dsTC015921R | TAA TAC GAC TCA CTA TAG GGCTAAATACCGGAAACGGCTCTAA |
| dsTC034643F | TAA TAC GAC TCA CTA TAG TGTTAGAGGCGGGTGAATTG |
| dsTC034643R | TAA TAC GAC TCA CTA TAG TCGCTCCTTCGTTGTTTACTT |
| dsTC010378F | TAA TAC GAC TCA CTA TAG GGAGCACCAGCAGGTTCTATTC |
| dsTC010378R | TAA TAC GAC TCA CTA TAG GGAGCCTTGACTTTCCTCCTTTC |
| dsTC008846F | TAA TAC GAC TCA CTA TAG GGATGGAAACGTAGCGACACTG |
| dsTC008846R | TAA TAC GAC TCA CTA TAG GGTGCAGTATTCTTCAGCCTTCTC |
| dsTC000126F | TAA TAC GAC TCA CTA TAG GGCGGCTACAAACATCAAGACATAAC |
| dsTC000126R | TAA TAC GAC TCA CTA TAG GGCTCGCTACGACCAGTTTCAATA |
| dsTC007435F | TAA TAC GAC TCA CTA TAG GGAGTCCGAGTCCGGAGAATTTA |
| dsTC007435R | TAA TAC GAC TCA CTA TAG GGGTGCCCTCGTAACCATTGATAG |
| dsTC013490F | TAA TAC GAC TCA CTA TAG GGGACGGGTTTGAGACCGATAAT |
| dsTC013490R | TAA TAC GAC TCA CTA TAG GGGTTCCAGGAGAGAACGTGAAA |
| dsTC008190F | TAA TAC GAC TCA CTA TAG GAGCTTCCTGGATCGGTTATG |
| dsTC008190R | TAA TAC GAC TCA CTA TAG CAGCGACCTCCTCATGTATTT |
| dsTC015420F | TAA TAC GAC TCA CTA TAG GAGAGTGTAGACAGGTGGGATA |
| dsTC015420R | TAA TAC GAC TCA CTA TAG GACAATTGGAGGGCACATAGA |
| dsTC004679F | TAA TAC GAC TCA CTA TAG GACGCTGGGATGGTTTGTAA |
| dsTC004679R | TAA TAC GAC TCA CTA TAG GTTCGGAGATGTCAGGATTGAG |
| dsTC008128F | TAA TAC GAC TCA CTA TAG TCCGCCTATTAGTCCAGGTTA |
| dsTC008128R | TAA TAC GAC TCA CTA TAG GGATTCTGAGGGACTTGTTGAG |
| dsTcART1 F | TAATACGACTCACTATAGGGAATAACCATGTCAGCGAGGAG |
| dsTcART1 R | TAATACGACTCACTATAGGGCTCCTCGACTTTGCCCTTAAT |
| **qPCR** | |
| qTcART1 F | GAAGGAGGATTTGGAGTTCTGT |
| qTcART1 R | CCGATACGTTTGTGGCATTTC |
| qTC008142F | CGAATGGGTGACCGAAGAA |
| qTC008142R | GGAGTTGAGACTCCAGTGTTAC |
| qTC006477F | GTCTAGTATGAAACTGGCCTCTC |
| qTC006477R | TTGGTGGGTTTGGACAGTAG |
| qTC011945F | CCTGACTTTGCCACCCTATC |
| qTC011945R | CCGATCTTTCCCTCGACTTTC |
| qTC003573F | CCTTATCAGAGGCAAGGCTAC |
| qTC003573R | CCCAACTCCAGTAACTCCTATAAC |
| qTC003198F | AGCCTATGCCACAACCTATG |
| qTC003198R | TGTGATGTGGCCTGGATTATT |
| qTC009742F | AAGCCGTAGTCACAGTAAACC |
| qTC009742R | CTGATTCCCTCTTGCACTCTC |
| qTC002077F | CGGAATTGTGACCGAACCTATC |
| qTC002077R | CACAGGCCACTCCAAACTATC |
| qTC014774F | GTTGCGAGCACCCTTTAGA |
| qTC014774R | GACTCATTGCCGTACCCATATT |
| qTC002507F | GTTACCGACAGAGGAGGAAATC |
| qTC002507R | CCAGCATCCTTGAGCCTTT |
| qTC004103F | ACTCGCGAAGTCGGAATAAC |
| qTC004103R | AGAGGCCGATTAGAATGTCTTG |
| qTC009267F | GCTTTAGCAACGCGTTTAGG |
| qTC009267R | GCTGCTGTAAGGTGAGAGAAT |
| qTC008518F | CGCGATGTGCTTCGTAGTAA |
| qTC008518R | CGACCTCAGATGCGCTAAAT |
| qTC009619F | CAGGGCTGTACCAAGGTTATT |
| qTC009619R | GGTTGTAGGCTCGTCGTTC |
| qTC000030F | TCCTCTGCCTCGTCGTAAA |
| qTC000030R | ATGAACCGGAATTGGCTCAG |
| qTC007251F | CTTTAACTGAGACCTACGGACTG |
| qTC007251R | GCCCATAATTTCGCCACTTG |
| qTC008129F | GGTGAACGGACTAGTGAACAAT |
| qTC008129R | TGTGGTTTAGGTTGCGTACTT |
| qTC015921F | CCATCATCACCACCCATTGA |
| qTC015921R | GGGCTTCGTCTTAGAAATCCA |
| qTC034643F | AAGAATAGAACCGGCAGAC |
| qTC034643R | TTTACTCAGCGTTTCCGT |
| qTC010378F | CATGTGTGGGAAAGTGTTGAG |
| qTC010378R | CGTGCTGAGTGTTGGATTTG |
| qTC008846F | TGTCAACTAAAGGGCAAGAAGG |
| qTC008846R | GGCATGCAGTTACCGTATATAGAA |
| qTC000126F | GAAACTGGTCGTAGCGAGATT |
| qTC000126R | CCTCATAATCGCCAACTGTACT |
| qTC007435F | CTGGGTACGTCCCTGTTTATTT |
| qTC007435R | TCTCCTGAAATCGCTCCAATAC |
| qTC013490F | TGACGTCATACCTTATCTCTGTT |
| qTC013490R | AGACTTGGATCATATCCTCCTTT |
| qTC008190F | CGGAACAAGAGAGTGCTCAA |
| qTC008190R | GGTGCATCCGCTTCTACAT |
| qTC015420F | TGGTTCGGCTGGCAAATA |
| qTC015420R | CGCCAGATCACTCGAAACA |
| qTC004679F | CGCGACCTTTCGCTAGTT |
| qTC004679R | CTCGTTCAGCCTCATCTTGTAG |
| qTC008128F | GGTACAATTGAGGAACCAAGGA |
| qTC008128R | GCCTGCATTGTGCTGATTTAG |
| **ChIP assay** | |
| TcE74_Promo F | GTCAAGGGTAAGTCCCGAAAT |
| TcE74_Promo R | TCGAACGCTTACTGAGGTAAAG |
| TcHR3-Promo F | ACTACGGTAGACCAGACAAG |
| TcHR3-Promo R | AGACCACGATAATGTTTCGTAT |
| TcE75-Promo F1 | GGTCATAGGCAACCAGCAATA |
| TcE75-Promo R1 | GGCGACACCGAACAATACA |
| TcEcR-Promo F | CAACCGCATTATTCAGGTCTTTC |
| TcEcR-Promo R  TcHSP90-Promo F  TcHSP90-Prmo R | CCGCAAATGTCAGCTTCTTG  GCGCTAAGTGAAGAGCTAAGA  ATGCACACACGAACAAATCAC |
| **EcREs cloning** | |
| EcRE-TcE75 F | ACCAGGGTACCCATGGCTGTGTTGAACTGT |
| EcRE-TcE75 R | GCTGCTACTCGCTCGAGCTGGCATTCC |
| EcRE-TcHR3 F | ACAAGGGTACCGATGATGACAACAACTGTC |
| EcRE-TcHR3 R | TACGCTCTCGAGTTATGCATTATGCAGGC |
| **Gene Cloning** | |
| TcNAA40 WT_F | ATCATTCCATGGATGGGGCGGAAAAGTTC |
| TcNAA40 WT_R | GACAGCAAGCTTTTACGACATCAATGGGTGG |
| TcNAA40 Mut-F | AAATTCATGATGCAAATTTTAGAACTG |
| TcNAA40 Mut-R | AATTGCTGGTTCCAACTGTAG |
| NAA40_SP F | AAGGAGTGGGGGAACCATGGCATGGGGCGGAAAAGTTCGGC |
| NAA40_W/O SP F | GTTAAAAAGGAGTGGGGGAACCATGGCATGCAAAAAATTCTGCAAGCGATG |
| NAA40 EGFP F | TCACCACCCATTGATGTCGATGGTGAGCAAGGGCGAGGAGC |
| NAA40 EGFP R | GCTCCTCGCCCTTGCTCACCATCGACATCAATGGGTGGTGA |
| EGFP-pIEx4 R | CCAGAAGATGCGGCCGCAAGCTTTAAGATACATTGATGAGTTTG |

**Note**: Primer names starting with ‘ds’ were used for dsRNA synthesis. Primer names containing ‘q’ were used for RT-qPCR analysis. Primer names containing ‘Promo’ were used for ChIP assay. Primer names containing “WT” were used for cloning wildtype NAA40, and Primer names containing “Mut” were used for making Actetyl-CoA binding motif lacking mutant NAA40 construct. Primers names containing “SP” were used for amplifying the complete ORF of NAA40 which contain predicted signal peptide at its N-terminal region. Primers names containing “W/O SP” were used for amplifying signal peptide lacking NAA40.

**
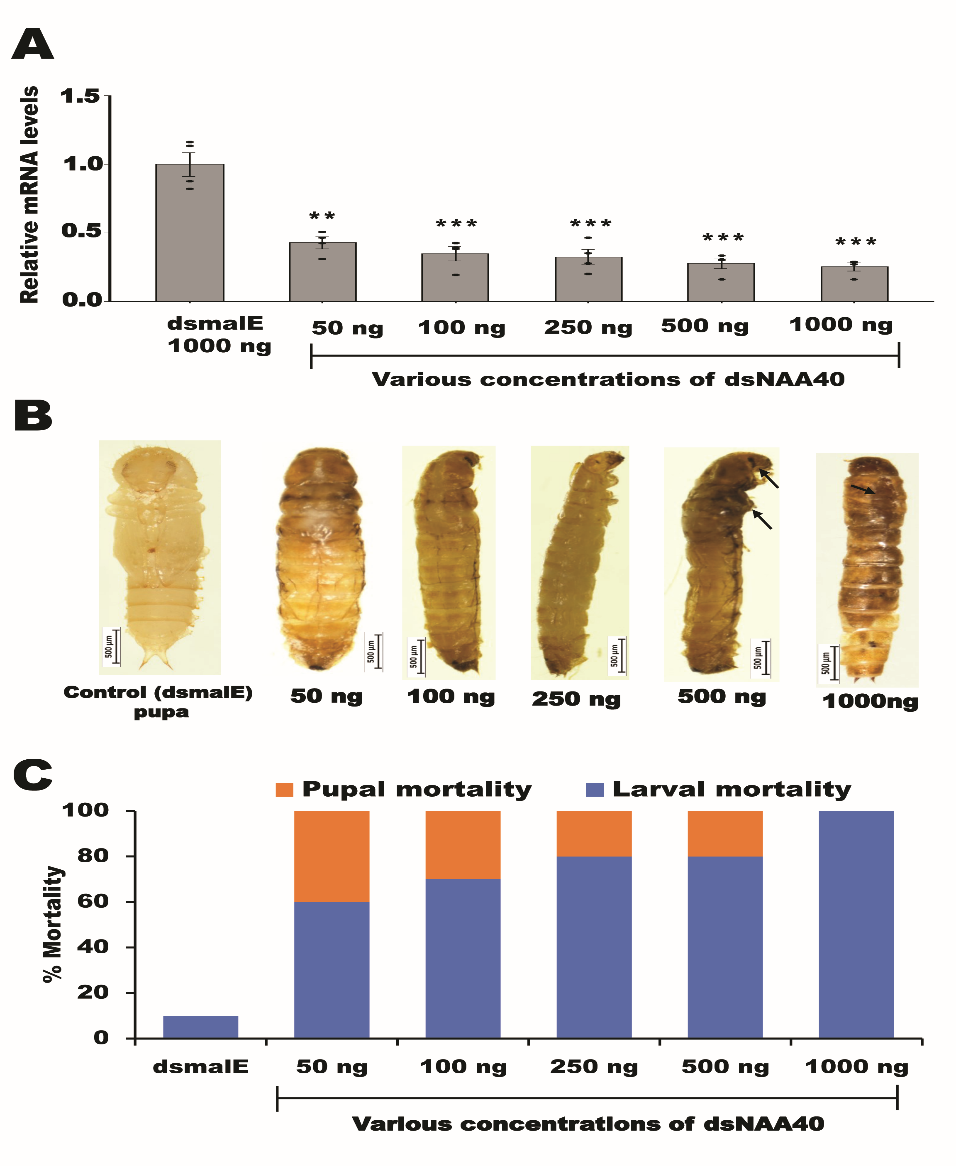
**

**Supplementary Fig. 1. Effect of different concentrations of *dsNAA40* on target gene knockdown, mortality and larval phenotype in *T. castaneum*. A)** The knockdown levels of *NAA40* varied across different concentrations of *dsNAA40*-injected larvae. Newly molted last instar larvae were injected with varying concentrations - 50, 100, 250, 500 and 1000 ng of *dsNAA40*. Control larvae were injected with dsRNA targeting the gene encoding maltose-binding protein from *Escherichia coli* (malE). The results presented as Mean±SE (n=4), reveal significant differences (****P*< 0.001; ***P*<0.01) in *NAA40* knockdown levels among various concentrations of *dsNAA40* treatments compared to the control treated with *dsmalE*, analyzed using One-way ANOVA. **B)** The impact of different concentrations of *dsNAA40* on larval phenotypes. Severe developmental defects, including larval-pupal intermediate phenotypes with compound eyes were observed in larvae injected with 500 ng and 1000 ng of *dsNAA40*. While lower concentrations - 50, 100, 250 ng of *dsNAA40* injection resulted in moderate phenotypes. Scale bar: 500 µM. **C)** Larval and pupal mortality varied among various concentrations of *dsNAA40* treatments.


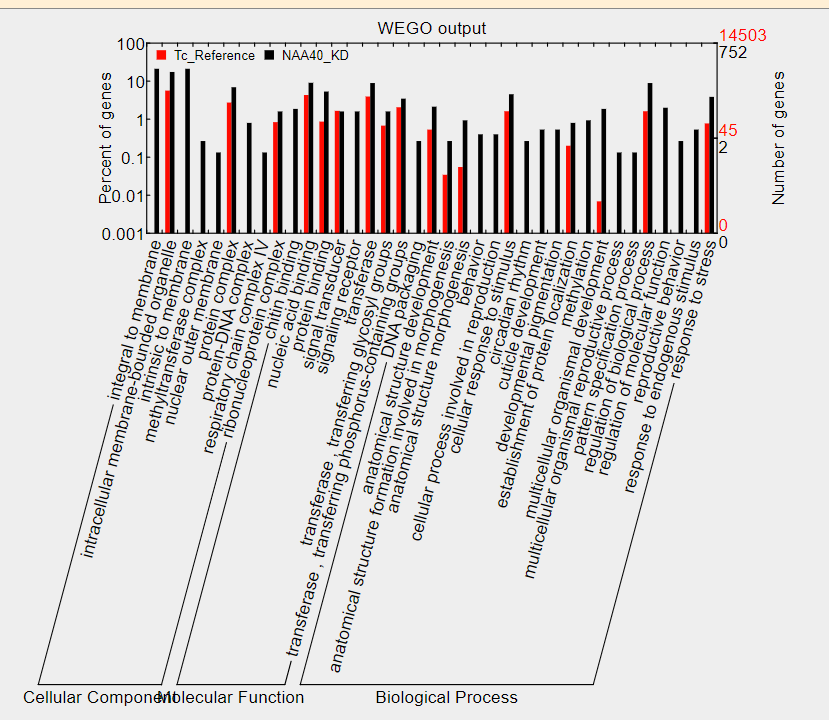


**Supplementary Fig. 2. WEGO plot depicts the enriched gene ontologies (GOs) in *NAA40* knockdown RNA-seq data.** Gene ontology (GO) enrichment analysis was done using the Web Gene Ontology Annotation Plot (WEGO), by plotting of the GO information of the differentially expressed genes (at FDR-corrected *P* < 0.05) against the GO terms of *T. castaneum* genome. GO terms for differentially expressed genes in *NAA40* knockdown larval transcriptome samples were obtained after Blast2Go analysis. GO categories are classified into cellular components, molecular function and biological process.


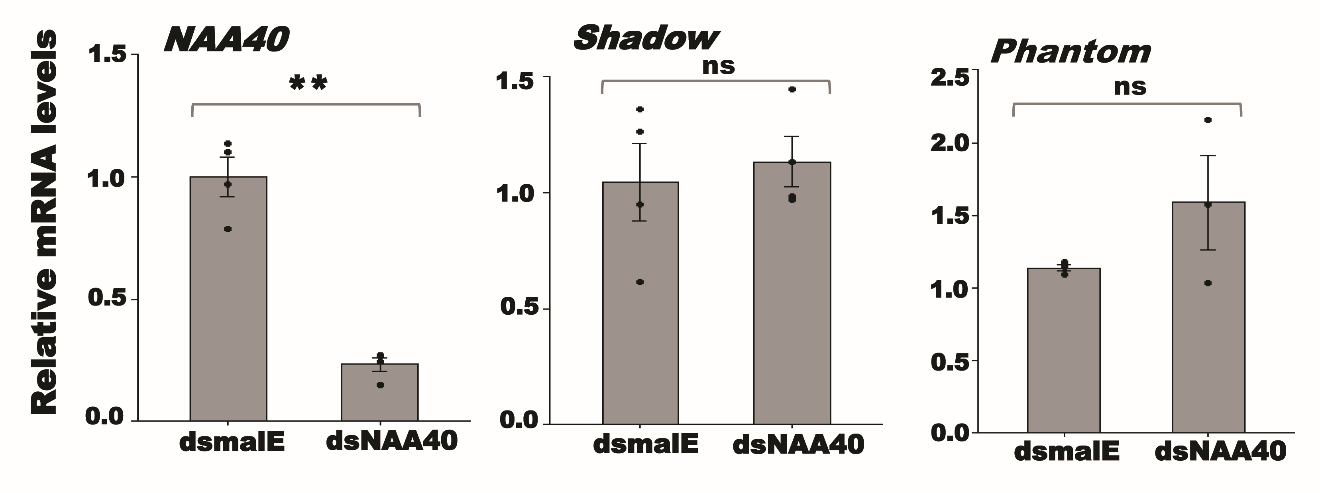
**Supplementary Fig. 3. Effect of *NAA40* knockdown on genes involved in ecdysteroid biosynthesis in *T. castaneum* larvae. A)** Knockdown of *NAA40* had no effect on the expression levels of *Shadow* and *Phantom* genes involved in ecdysteroids biosynthesis. Newly molted last instar larvae injected with 1 µg of *dsNAA40*, while control larvae injected with *dsmalE*. The results presented as Mean±SE (n=4) and data analyzed using One-way ANOVA. Here, ***P*<0.01 inidcates significant differences in the *NAA40* expression levels in *dsNAA40* treatment compared to the control treated with *dsmalE*; ns, not significant.

**
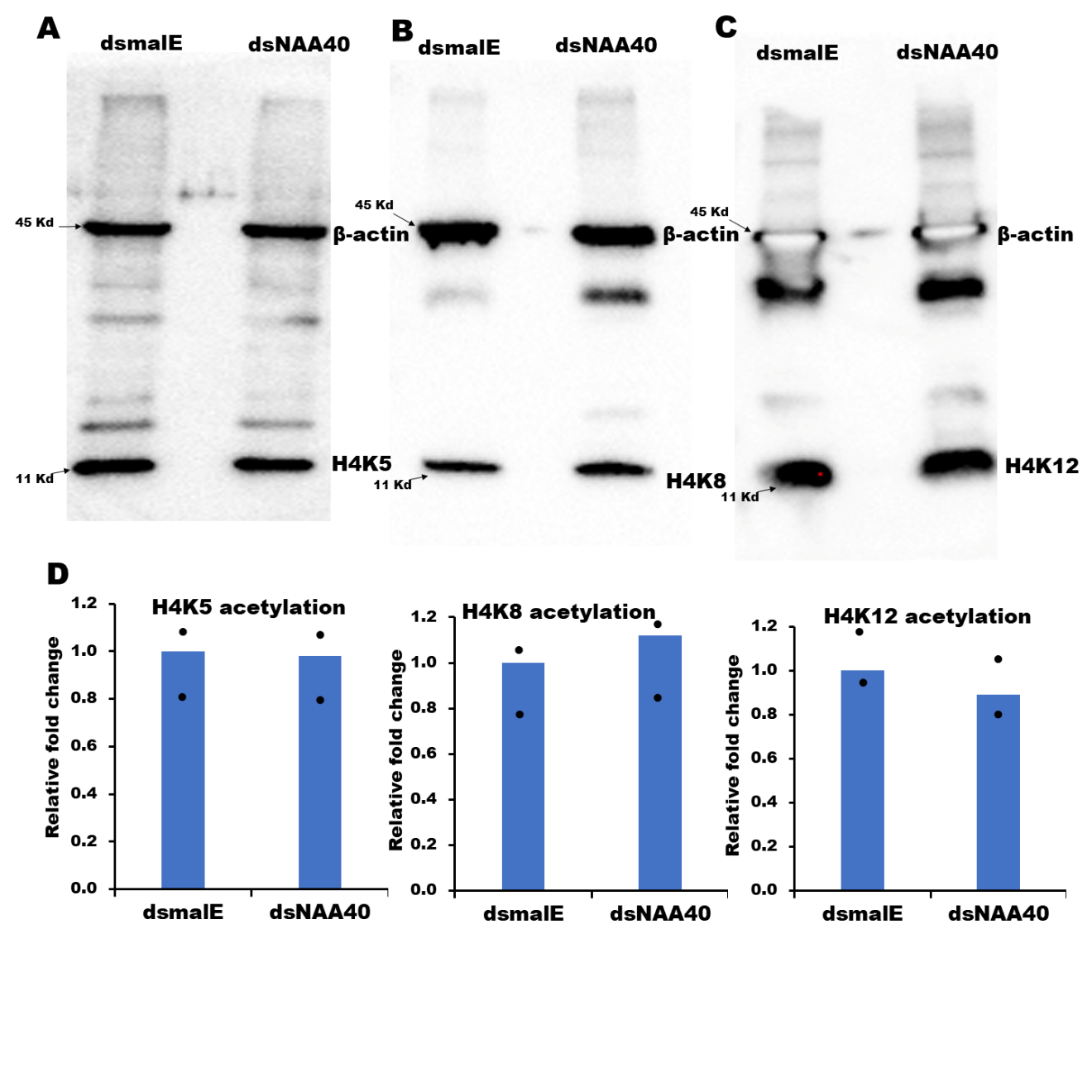
**

**Supplementary Fig. 4**. *NAA40* knockdown had no effect on acetylation levels of Lys5, Lys8 and Lys12 residues in the N-terminal tail of histone H4. **A)**Acetylation levels of Lys5 of histone H4 in the *NAA40*knockdown TcA cells. **B)**Acetylation levels of Lys8 of histone H4 in the *NAA40* knockdown TcA cells. **C)**Acetylation levels of Lys12 of histone H4 in the *NAA40* knockdown TcA cells. **D)**Quantification of western blot band intensities shows that no significant differences in the acetylation levels of Lys5, Lys8 and Lys12 residues of histone H4 were observed in the *NAA40* knockdown cells compared to their levels in control cells treated with *dsmalE*. Band intensities from duplicate blots were measured using the Image J software. The mean band intensity of target protein acetylation between the treatment (*dsNAA40*) and control (*dsmalE*) was normalized using the loading control protein, β-Actin. Normalized protein acetylation levels were then represented as relative fold change compared to the control treated with *dsmalE*.**
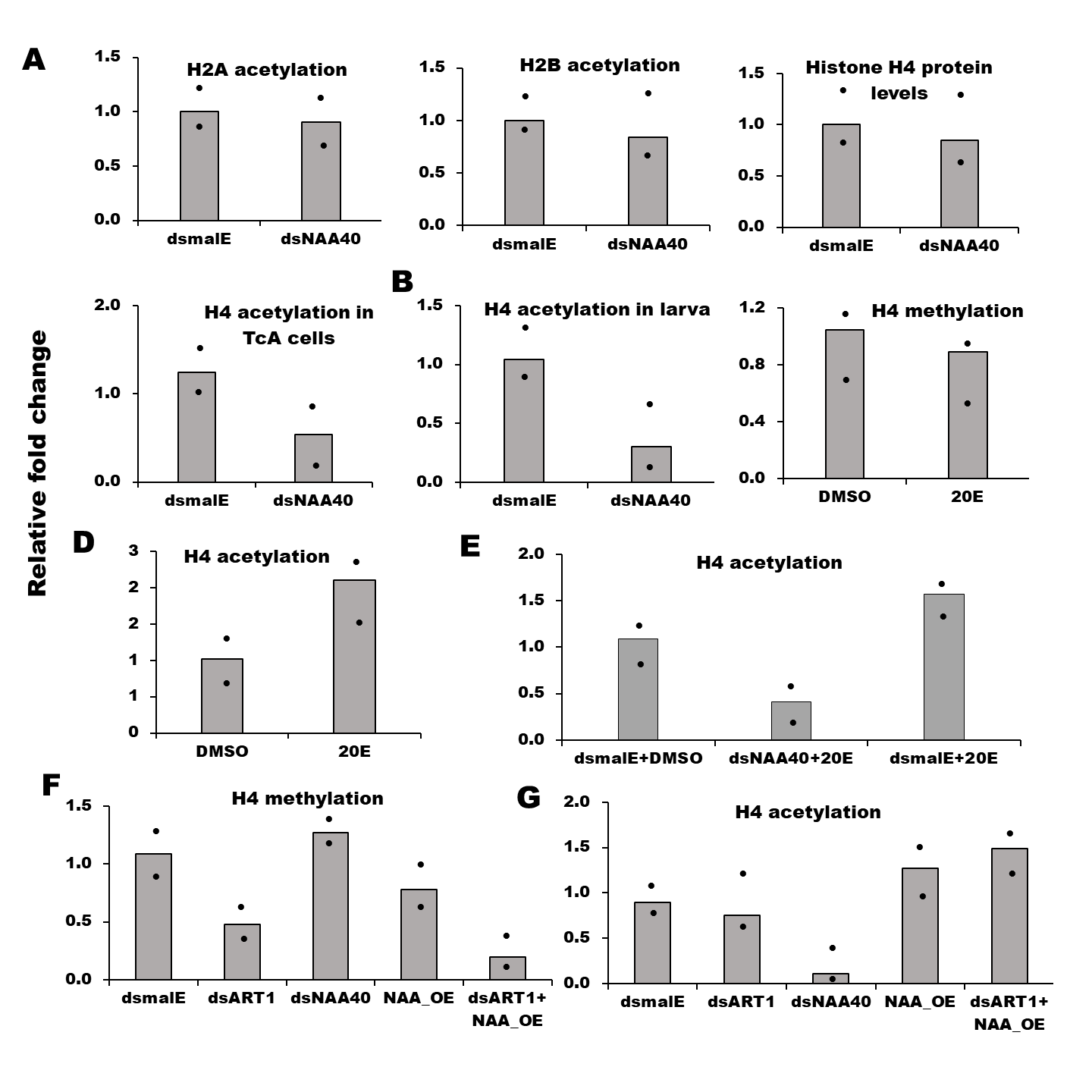
Supplementary Fig. 5**. Histone acetylation levels in TcA cells or *T. castaneum* larvae subjected to knockdown or overexpression of *NAA40* or *ART1*. Western blot analysis was performed on proteins extracted from TcA cells or *T. castaneum* larvae after *dsNAA40* or *dsmalE* treatments or overexpression of *NAA40* followed by hormone treatments. Band intensities from duplicate blots were measured using the Image J Lab software (Bio-Rad, USA). The mean band intensity of target protein acetylation/methylation among treatments and control was normalized using the loading control protein, β-Actin. Normalized protein acetylation/methylation levels were then represented as relative fold change compared to the control (left bar in the each figure). **A**) Acetylation levels of histone H2A, H2B and H4, or histone H4 protein levels in TcA cells exposed to *dsNAA40* or *dsmalE*. **B**) Histone H4 acetylation in *T. castaneum* larvae treated with *dsNAA40* or *dsmalE*. **C**) Histone H4 Arginine 3 methylation levels in 20-hydroxyecdysone (20E) treated TcA cells. **D**) Histone H4 acetylation levels in 20E treated TcA cells. **E**) *NAA40* knockdown prevents 20E-mediated induction of the histone H4 acetylation in TcA cells. **F**) Histone H4 Arginine 3 methylation levels in *ART1*, *NAA40* knockdown, *NAA40* overexpressed alone or along with *ART1* knockdown in TcA cells. **G**) Histone H4 acetylation levels in *ART1*, *NAA40* knockdown, *NAA40* overexpressed alone or in combination with *ART1* knockdown in TcA cells.

**
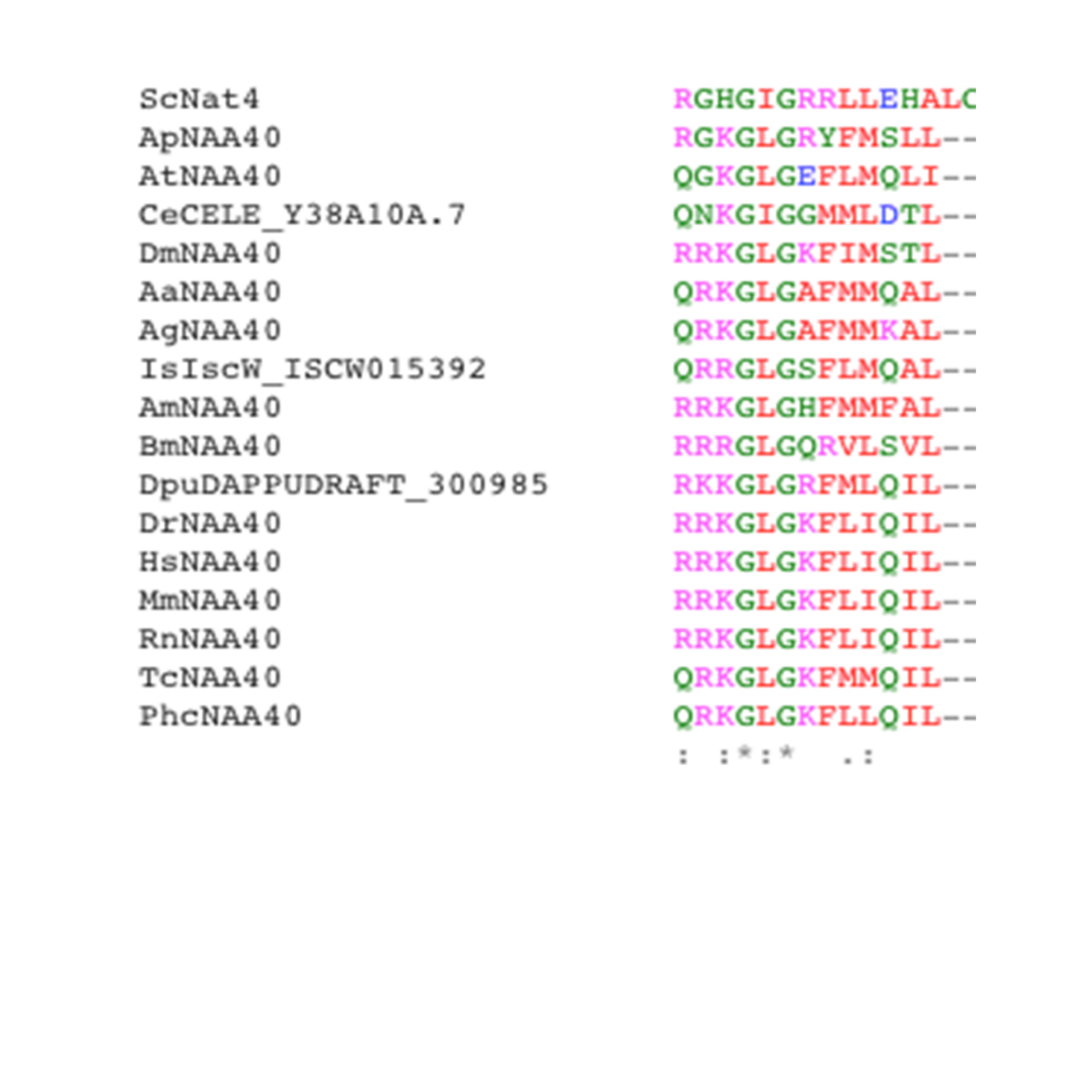
**

**Supplementary Fig. 6**. Acetyl CoA binding domain of *NAA40* is conserved across the species. Acetyl CoA binding domain from different species aligned using Clustal2.1. Here, **Fungi:** *Saccharomyces cerevisiae* (Sc) (Yeast). **Plantae:** *Arabidopsis thaliana* (At)**. Nematoda**: *Caenorhabditis elegans* (Ce). **Arthropoda:** *Acyrthosiphon pisum* (Ap) (Insecta: Homoptera), *Drosophila melanogaster* (Dm) (Insecta: Diptera), *Aedes aegypti* (Aa) (Insecta: Diptera), *Anopheles gambiae* (Ag) (Insecta: Diptera), *Ixodes scapularis* (Is) (Arachnida: Ixodida), *Apis mellifera* (Am) (Insecta: Hymenoptera), *Bombyx mori* (Bm) (Insecta: Lepidoptera), *Daphnia pulex* (Dp) (Crustacea: Water flea), *Pediculus humanus capitis* (Phc) (Insecta: Phthiraptera), *Tribolium castaneum* (Tc) (Insecta: Coleoptera), **Vertebrata:** *Homo sapiens* (Hs), *Danio rerio* (Dr), *Rattus norvegicus* (Rn), *Mus musculus* (Mm).

**
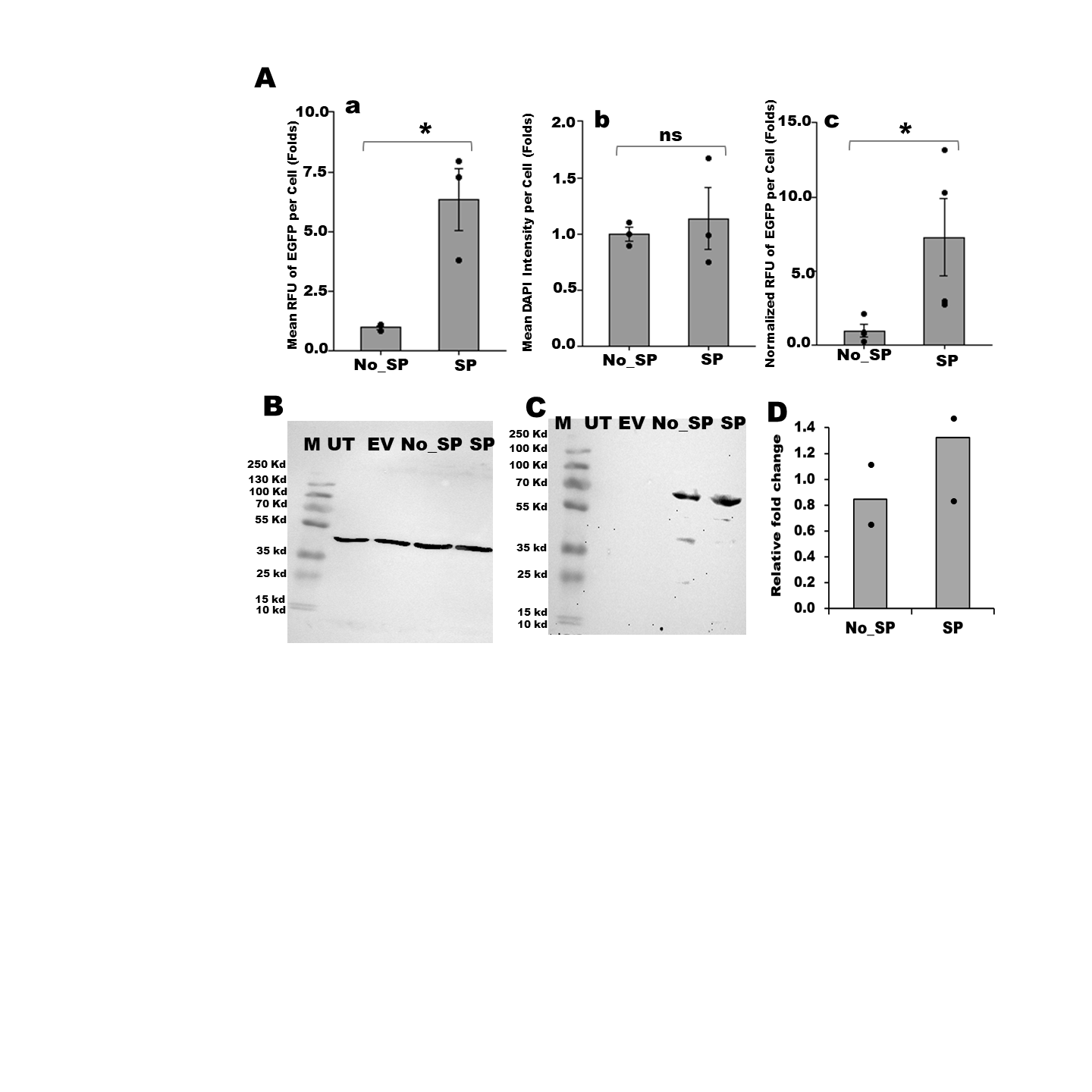
Supplementary Fig. 7.** Exogenously expressed wildtype *NAA40* localized into the nucleus. TCA cells were transfected with signal peptide having wildtype *NAA40-EGFP* or N-terminal signal peptide lacking *NAA40-EGFP* fusion constructs. At 72 h post-transfection, cells were mounted using the DAPI nuclear staining and imaged under a Confocal microscope at 63X magnification using blue and GFP filters. DAPI and GFP fluorescence intensities of four (N=3) images were quantified using Image J software. **a**) Relative Fluorescence Units (RFU) of EGFP per cell in N-terminal signal peptide lacking *NAA40-EGFP* (No_SP) and wildtype *NAA40-EGFP (*with signal peptide) (SP) constructs transfected cells. **b**) Mean DAPI intensities in N-terminal signal peptide lacking *NAA40-EGFP* (No_SP) and wildtype *NAA40-EGFP (*with signal peptide) (SP) constructs transfected cells. Here, ns: non-significant. **c**) After normalizing with DAPI, the mean normalized RFU of EGFP signal in N-terminal signal peptide lacking *NAA40-EGFP* (No_SP) and wildtype *NAA40-EGFP (*with signal peptide) (SP) constructs transfected cells. ‘*’ denotes the significant differences in the mean fluorescence intensity between the N-terminal signal peptide lacking *NAA40-EGFP* and wildtype *NAA40-EGFP (*with signal peptide) fusion constructs transfected cells at *P <* 0.05 analyzed using the One-way ANOVA. **B**) β-actin protein levels in un-transfected (UT) TcA cells and cells transfected with an empty vector control (EV) or wildtype *NAA40-EGFP* (SP) or N-terminal signal peptide lacking *NAA40-EGFP* fusion constructs (No_SP). **C**) NAA40 protein levels in un-transfected (UT) TcA cells and cells transfected with an empty vector control (EV) or wildtype *NAA40-EGFP* (SP) or N-terminal signal peptide lacking *NAA40-EGFP* fusion constructs (No_SP). **D**) Quantification of western blot band intensities shows that no significant differences in Wildtype *NAA40-EGFP* or N-terminal signal peptide lacking *NAA40-EGFP* fusion proteins. Band intensities from western blot were measured using the Image J software and represented as a fold change compared to N-terminal signal peptide lacking *NAA40-EGFP* fusion protein at 95% CI (confidence interval) analyzed using one-way ANOVA.


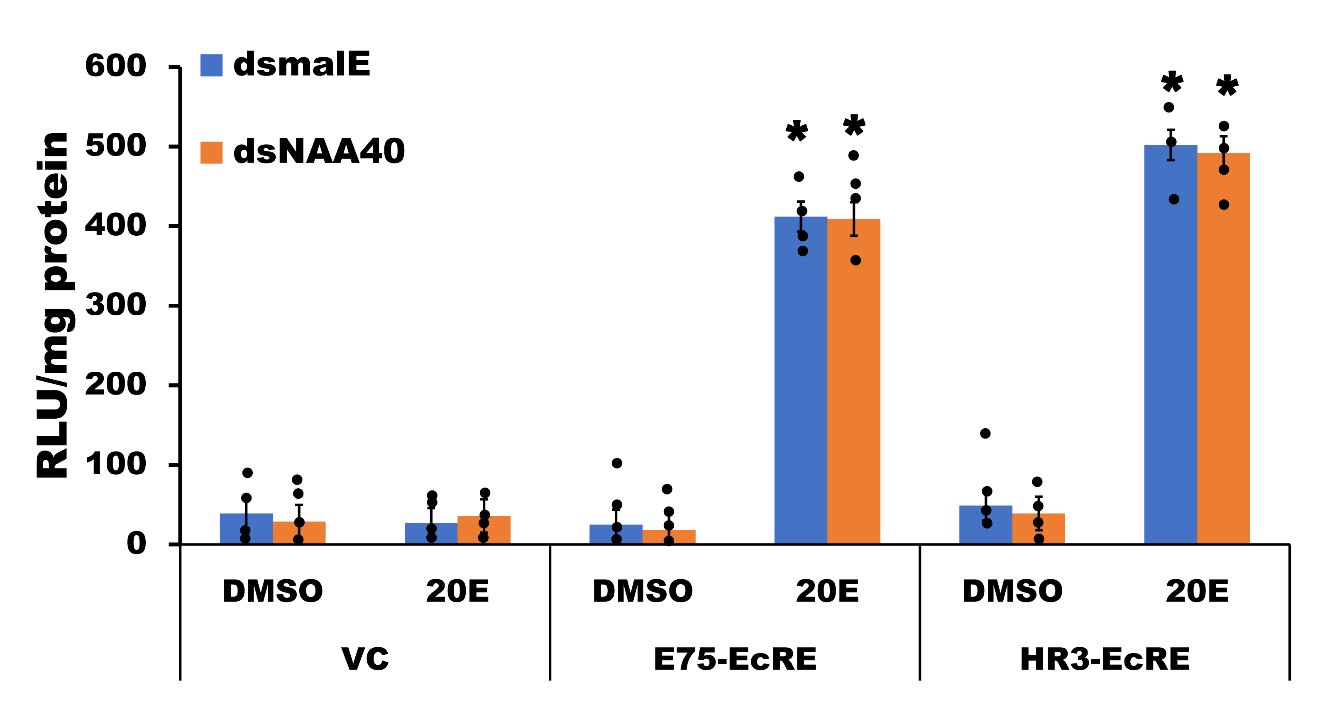


**Supplementary Fig. 8**. *NAA40* knockdown does not affect the luciferase activity driven by EcREs of *E75* and *HR3* promoters. TcA cells were transfected with the luciferase reporter constructs where the luciferase gene is under the control of *E75-* and *HR3-e*cdysone response elements (EcREs) containing promoters. The transfected cells were treated with *dsmalE* or *dsNAA40* for 48 h and exposed to DMSO or 20E for an additional 24 h and the luciferase activity was measured. The mean ±SE (N=4) is shown. ‘*’ denotes significant differences in the luciferase activity levels between the 20E exposed cells compared to control cells exposed to DMSO at *P*<0.005, analyzed using the One-way ANOVA. No significant differences were observed between *dsmalE* and *dsNAA40* treated cells. Here, DMSO, Dimethyl sulfoxide; 20E: 20-hydroxyecdysone. VC: Vector control; RLU: Relative luminescence units.


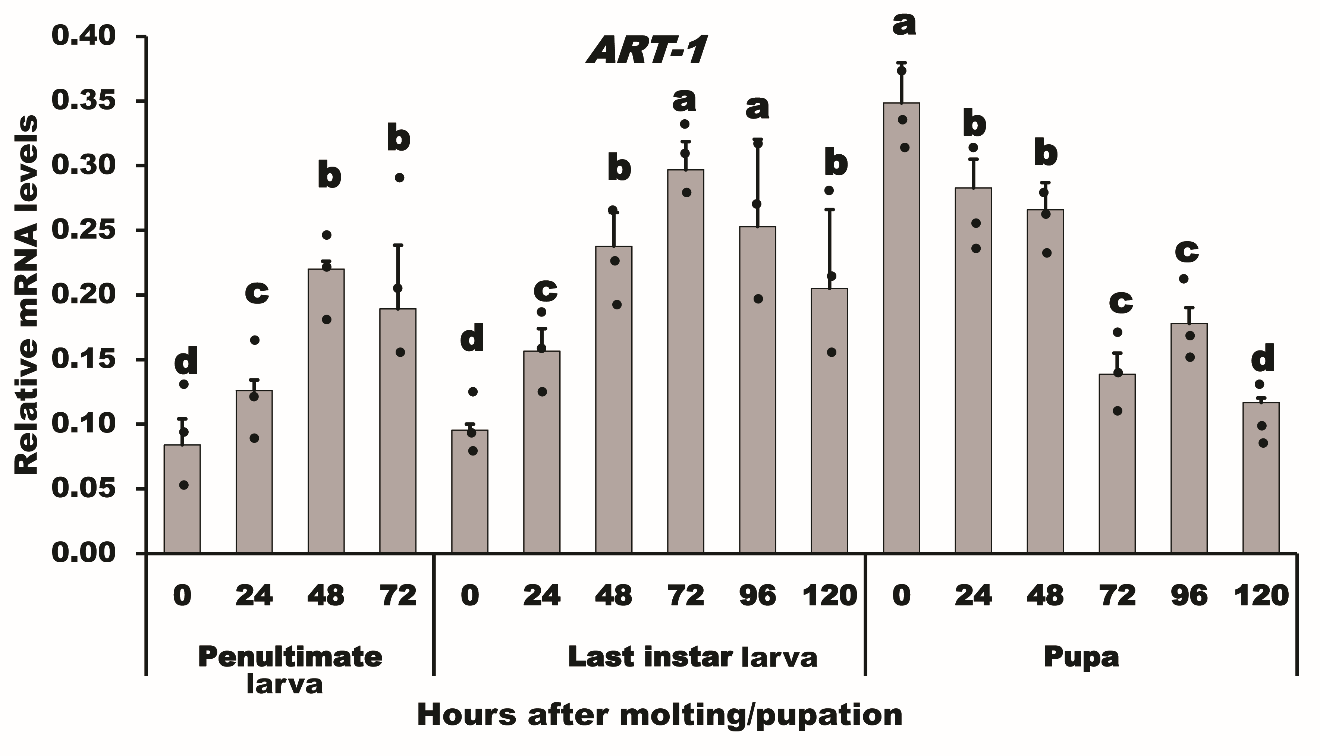
**Supplementary Fig. 9**. Developmental expression profile of *ART-1*in *T. castaneum.*RNA samples were collected at 24 h intervals during the penultimate, last instar larval, and pupal stages. For each time point, two larvae/pupae were used for one replication and total three replicates (N=6). *ART-1* mRNA levels  were determined by RT-qPCR. The ribosomal Protein 49 (*RP49*) mRNA levels were used for normalization. Bars with the same letters are not significantly different from each other at *P <* 0.05 analyzed using ANOVA with Post hoc Tukey HSD test.

**
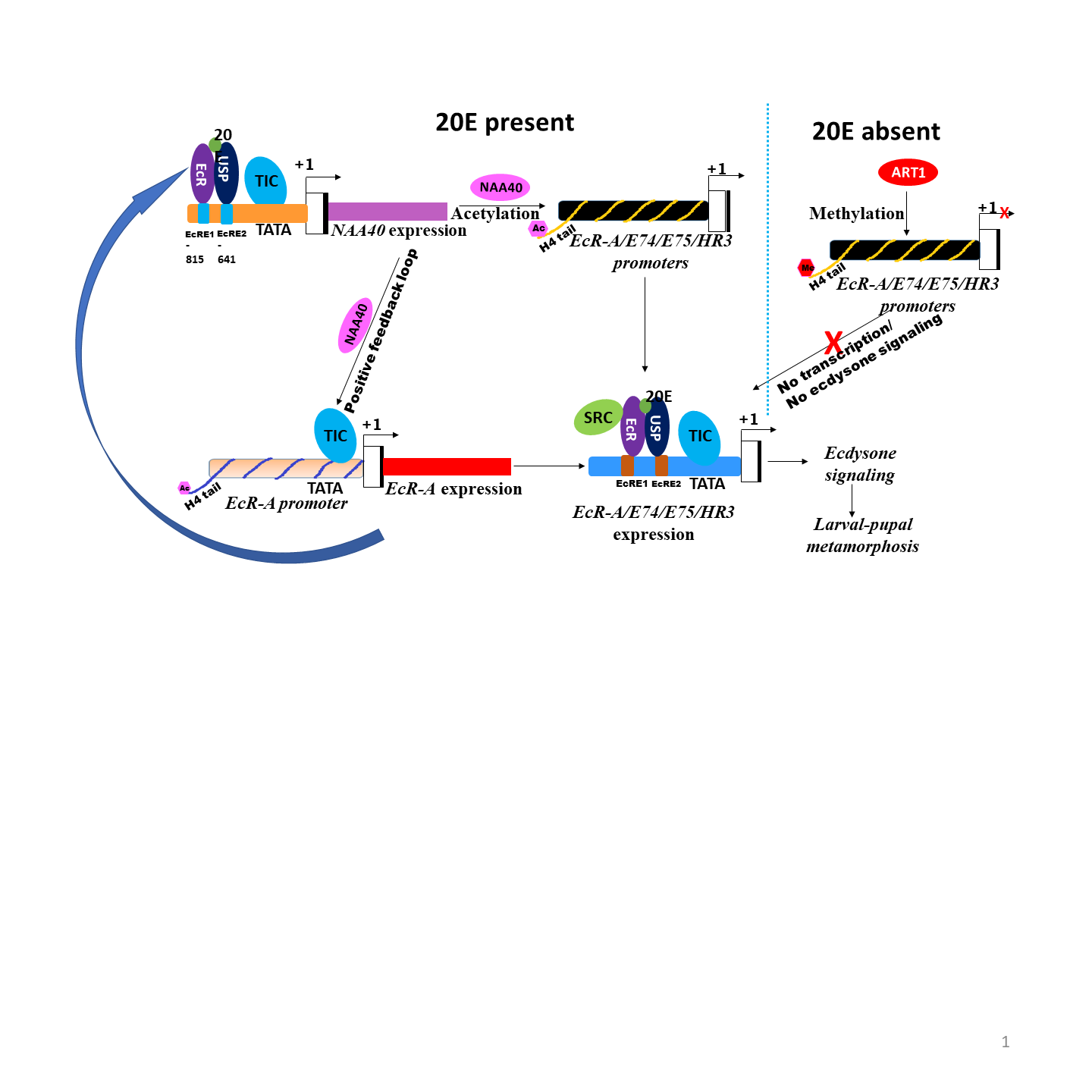
**

**Supplementary Fig. 10. A model for the NAA40 mediated regulation of ecdysone signaling in *T. castaneum*.** During the larval-pupal metamorphosis, ecdysteroids (20-hydroxyecdysone, 20E is the most active form) induce the *NAA40* gene expression perhaps by promoting the interaction of ecdysone receptor complex EcR/USP with the predicted putative ecdysone response elements (EcREs) in the promoter of *NAA40*. NAA40 regulates its own activator, *EcR-A* expression through positive feedback regulatory mechanisms. NAA40 acetylates histone H4 localized at the promoters of the key ecdysone response genes, *EcR*, *E74*, *E75*, and *HR3*. Acetylation of histone H4 at the promoters of these genes may facilitate the recruitment of the ecdysone receptor complex along with co-activators such as SRC/CBP to the above gene promoters, thereby inducing their expression. The induction of ecdysone response genes promotes larval-pupal metamorphosis. While in the absence of ecdysone, ART1 methylates histone H4 may be localized at the promoters of *EcR*, *E74*, *E75*, and *HR3* and prevents the expression of these genes. During the larval-pupal metamorphosis, NAA40 enhances the acetylation of histone H4, leading to a reduction in ART1-mediated methylation and the expression of ecdysone response genes associated with metamorphosis in *T. castaneum*. 20E, 20-hydroxyecdysone; EcR/USP, Ecdysone receptor/Ultraspiracle protein- form heterodimer ecdysone receptor complex; SRC, steroid-receptor co-activator and EcRE: Ecdysone response elements; TIC, Transcription initiation complex.

**Supplementary Fig. 11.**

Unprocessed/uncropped western blot images for Figure 4.


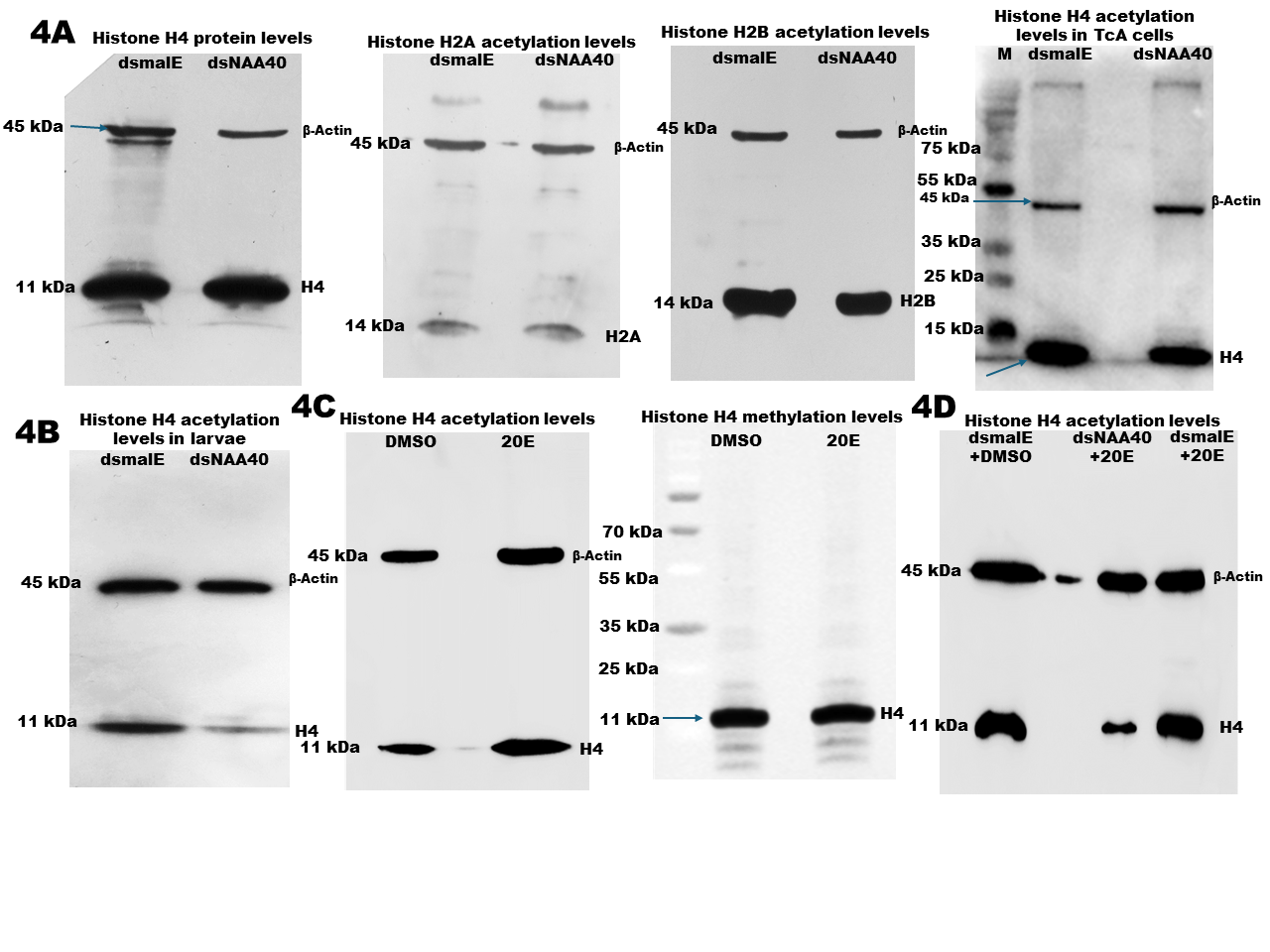


**Supplementary Fig. 12**

Unprocessed/uncropped western blot images for Figure 8C


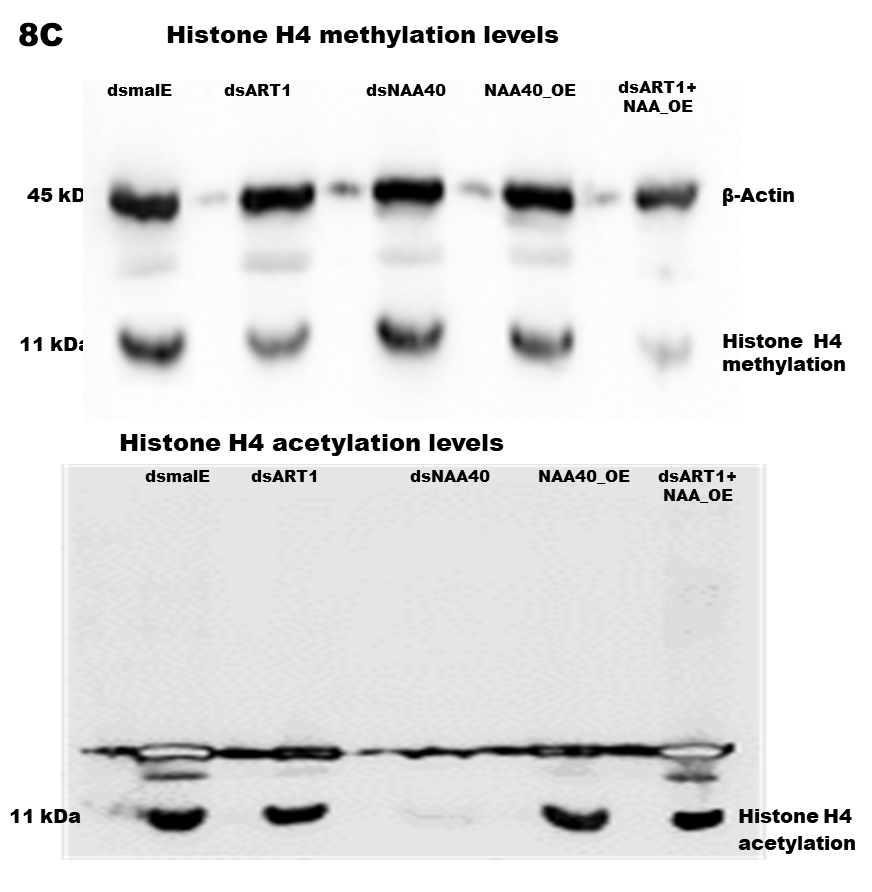


45 KDa

11 KDa
