# Supplementary material for: N(alpha)-acetyltransferase 40-mediated histone acetylation plays an important role in ecdysone regulation of metamorphosis in the red flour beetle, Tribolium castaneum
Source: Commun Biol. 2024 May 3;7:521. doi: 10.1038/s42003-024-06212-7 (PMC11068786; doi:10.1038/s42003-024-06212-7)
Supplement: Supplementary file 2 — Description of additional supplementary files [file 42003_2024_6212_MOESM2_ESM.docx]

Description of Additional Supplementary Files

**File name:** Supplementary data 1

**Description:** List of downregulated and upregulated genes in NAA40 knockdown animals.

**File name:** Supplementary data 2

**Description:** The source data behind the graphs in the paper
